# A benchmark study of force fields implemented in CSD software

**DOI:** 10.1007/s11224-025-02715-z

**Published:** 2026-01-10

**Authors:** Lily M. Hunnisett, Pietro Sacchi, Andrew G. P. Maloney

**Affiliations:** The Cambridge Crystallographic Data Centre, 12 Union Road, Cambridge, CB2 1EZ UK

**Keywords:** Force field, Lattice energy, Polymorph, Benchmark

## Abstract

We have performed a benchmarking study to assess the performance of the CLP, UNI, CSD-OPCS16, DreidingII and Momany force fields available in the CSD Software. Our evaluation focused on the reproduction of experimental sublimation enthalpies for a large dataset of 664 crystal structures from the Cambridge Structural Database (CSD) and on the analysis of their crystal structures after optimisation with a new force field based optimiser. An analysis of our results for subsets of different chemical compound classes allowed for further insight on the performance of each force field. We have also assessed the ability of the presented force fields to reproduce known relative stabilities of polymorph families by comparison with DFT data. Our results show that, while all force field methods are able to reproduce sublimation enthalpies within the expected accuracies, overall errors are smaller for the UNI (11%), CSD-OPCS16 (13%) and CLP (13%) force fields. DreidingII and Momany have overall larger errors (22% and 26%, respectively), which can be notably reduced with the aid of structural optimisations. When looking at different classes of compounds, we found that no one force field would perform better than the other, and that accuracy is strictly dependent on the chemistry of the system of interest. The limitations of general purpose force fields in reproducing the small energy differences typical of polymorphs are also discussed. In particular, we found these methods to be rarely suitable for this task, and only reliable when the energy differences between polymorphs are larger than the uncertainties associated with force field methods. We have outlined our key findings as a source of guidance to aid the selection of force fields for energetic studies, and have suggested potential applications of these methods for further exploration.

**Supplementary Information:**

The online version contains supplementary material available at 10.1007/s11224-025-02715-z.

## Introduction

Benchmarking different force field methods against experimental data provides an essential resource for rationally choosing which is the most suitable to accurately model and predict properties of organic molecular materials [1–4].

It is well known that lattice energies of molecular crystals can be accurately calculated using *ab initio* quantum chemistry methods such as periodic Density Functional Theory (pDFT), post-Hartree Fock, or semi-empirical methods, but with high computational costs [1]. The high accuracy of such methods can be largely attributed to accounting for electron correlation in some form, in contrast to classical physics-based force field methods. Despite not capturing quantum effects or electron behaviour explicitly, force fields offer a faster and more efficient alternative and provide a reasonable degree of accuracy, especially when tailored to specific chemical systems via careful alteration of parameters. Force fields are often used in conjunction with geometry optimisation algorithms to quickly obtain a reasonable stable geometry with respect to the potential energy surface of a chemical system. This can then be used as a precursor for more accurate methods to significantly reduce the required computational resources. This is often the case in Crystal Structure Prediction (CSP) workflows, where force fields are utilised to estimate relative energies on large scales (tens of thousands of structures) to identify the theoretically most stable polymorphs. While it is currently common practice to incorporate a DFT-calculated intramolecular correction to account for molecular relaxation in the gas phase when calculating lattice energies of organic molecular crystals [4, 5], experimental sublimation enthalpies can still be reproduced within reasonable accuracy using intermolecular potentials [6] alone. For the above reasons, such potentials are also a versatile tool for application to a variety of methods, including the prediction of crystal morphologies [7, 8].

Assessing the accuracy of energy-based methods is challenging due to the inherent uncertainties in both theoretical approaches and experimental measurements used for benchmarking. In a critical analysis of sublimation enthalpies for 1665 compounds, Chickos and Gavezzotti concluded that an uncertainty of 5-10% can normally be associated with experimental determinations [6, 9] and that uncertainties in the same range are to be expected for calculated lattice energies. Even in the case of energies calculated with quantum-mechanical methods, which have reported errors of ± 3 kJ mol$^{-1}$ [10, 11], uncertainties remain similar to those of experimental measurements [12].

In an earlier work, Gavezzotti found the lattice energies of 154 compounds, calculated with the Coulomb-London-Pauli (CLP) force field, to be in *very good agreement* with experimental values, having an average error of 7.7% [13]. This increased to 12% after comparison with a larger set of 669 compounds [6]. Another work evaluating a selection of force fields for the calculation of lattice energies of 235 molecular crystals found COMPASSII [14] to perform best for pharmaceutically-relevant systems, with a root mean squared error of 17.4 kJ mol$^{-1}$ [15].

Despite the large associated uncertainties, benchmark studies against experimental sublimation energies remain a valuable exercise, which allows to identify trends and outliers to better aid the development of new calculation methods, but also to provide researchers interested in lattice energy calculations with valuable resources to find the calculation method best suited for their needs.

Multiple force fields are now available in the CSD Software: through the morphology prediction program *VisualHabit* (CSD-Particle) or the newly introduced crystal structure optimisation algorithm *Crystal Optimiser* (CSD-Materials). Here, we present an analysis of lattice energies calculated for optimised and unoptimised crystal structures from the dataset originally collected by Chickos and Gavezzotti. By comparing our results with sublimation enthalpies for different classes of compounds, we aim to provide general guidelines to aid the selection of the appropriate calculation method for the system of interest. In addition, we have calculated relative lattice energies for four model polymorphic systems (tolfenamic acid, Pfizer compound PF-06282999, ritonavir, and ROY), and we compare our results to state-of-the-art quantum mechanical (QM) methods.

## Methods

We have compared calculated lattice energies of optimised and unoptimised crystal structures against sublimation enthalpies for a dataset of 664 compounds with entries in the Cambridge Structural Database (CSD) using five force fields currently available in our software and which are described below. Their accuracy was also tested against known relative stabilities of four polymorphic systems.

### Datasets

The results presented in this work are based on calculations for two main datasets, which are briefly described here. A larger dataset based on the work of Chickos and Gavezzotti [6] (CG2019) was used for the statistical analysis of the performance of both lattice energy and crystal structure optimisation calculations by focusing on the comparison with experimental sublimation enthalpies. A much smaller dataset of polymorphic compounds was instead used to compare the results of *Crystal Optimiser* with state-of-the-art periodic-DFT calculations, and to determine whether the force fields currently available in CSD suites are able to correctly rank the stability of polymorphs of interest.

#### The CG2019 dataset

A dataset of 669 compounds with associated crystal structures available in the CSD was extracted from the CG2019 publication. The CSD refcodes assigned to each compound in the original publication were used for our calculations, except for six cases where they had to be updated. A small portion of structures was excluded from our final analysis due to unresolved problems (5 structures for UNI, CSD-OPCS16, DreidingII and Momany; 6 structures for CLP). In addition, 114 structures containing halogen atoms were not used for calculation with the Momany force field due to missing parameters for these atoms. Thus, the final number of structures from the CG2019 dataset that were analysed in this work were 664 for UNI, CSD-OPCS16 and DreidingII, 663 for CLP and 550 for Momany. Details about CSD refcode changes and calculation failures are reported in the Supporting Information. For details about the original dataset preparation, interested readers are invited to the original publication by Chickos and Gavezzotti [6].

#### Classification of types of compounds

Calculation results for the CG2019 dataset were also analysed based on chemical classification, where classes of compounds were assigned using the Isostar library of intermolecular interactions [16]. Isostar groups were found automatically for each structure and further clustered so that each compound could be assigned to one or more of 25 classes of compounds. Structures for which classification was not successful were grouped in a general *Other* class. As an example, benzoic acid (BENZAC20) was classified as *Arene* and *Carboxylic acid*, whereas 2-bromobenzoic acid (BRBZAC01) was classified as *Haloarene* and *Carboxylic acid*.

#### Polymorphic dataset

A small set of 27 crystal structures and associated energy ranking for four polymorph families were compared with periodic-DFT results. The selected compounds were tolfenamic acid (TFA, 9 structures, refcode family KAXXAI), Pfizer compound PF-06282999 (4 structures, refcode family XULBAL), ritonavir (2 structures, refcode family YIGPIO) and ROY (12 structures, refcode family QAXMEH). This choice of compounds was based on the availability of QM-calculated lattice energies and the observation of multiple polymorphs.

### Software

All calculations were performed using tools from the CSD-Materials and CSD-Particle suites. Mercury 2025.2 was used to run calculations on individual structures when required, while the CSD Python API version 3.5.0 was used to run batch calculations and to analyse results.

#### Available force fields

The force fields considered in this work are suitable for the majority of organic molecular crystals and their applicability will depend on the types of atoms present in the system of interest. CLP is an atom-atom potential parameterised using experimental sublimation enthalpies and includes parameters for H, C, N, O, S, F, Cl, Br, I, P, B, Si, As, Se [13, 17] as well as Li^+^, Na^+^, K^+^, Rb^+^, Cs^+^, Ca^2+^, F^-^, Cl^-^, Br^-^ and I^-^ ions [18]. The UNI force field is a semi-empirical set of intermolecular potentials designed for organic crystals and parameterised for H, C, N, O, F, Cl, Br, I, P, B, Si, and S [19, 20]. CSD-OPCS16 (Cambridge Structural Database Optimised Potential for Crystal Structures 2016) has parameters based on UNI, and was adapted to better reproduce structures found in the CSD. It was originally parameterised for the application to knowledge-based CSP methods [21]. DreidingII is a combined general force field designed for organic, biological, and main group inorganic molecules parameterised for H, C, N, O, F, P, S, Cl, Br, I, B and Si [22]. Finally, Momany is designed for hydrocarbons, carboxylic acids and amides and parameterised accordingly for H, C, N, O, and S [23]. The functional forms of these force fields are reported in the Supplementary Information.

#### Crystal structure optimisation

Crystal structures were optimised using the *Crystal Optimiser* module from CSD-Materials. In this module, the score for a crystal structure, $Z_{total}$, is minimised until convergence to a local minimum. This score is defined as:

$$Z_{total} = Z^{FF}_{inter} + \kappa Z^{KBF}_{intra}$$

$Z^{FF}_{inter}$ is an intermolecular stabilisation score calculated with one of the available force fields and that corresponds to the crystal lattice energy (see next section for more details). $Z^{KBF}_{intra}$ is an intramolecular score that accounts for the effect of changes of molecular geometries (bond lengths, bond angles and torsions) within the crystal structure of interest. This intramolecular score is calculated using a knowledge-based force field derived from the CSD [24] and needs to be scaled by a factor $\kappa$ which is calculated for each individual structure and was found to have average magnitudes of 0.03 and 0.09 for structures in the CG2019 and polymorphic datasets, respectively. We note that the intramolecular score cannot be directly associated with a stabilisation energy value, despite its contribution to the overall optimisation score and so the scores returned by *Crystal Optimiser* should not be properly considered as crystal lattice energies.

*Crystal Optimiser* uses an implementation of gradient-based quasi-Newton Limited Memory BFGS method [25] for its calculations. The automatic differentiation library CppAD [21, 26] is also used to increase calculation efficiency if either UNI, CLP and CSD-OPCS16 are selected as force field to calculate $Z^{FF}_{inter}$.

Several options are available in *Crystal Optimiser* to control the desired type of optimisation. In this work, we have explored two limiting combinations of these options:

- Keep the unit cell parameters fixed and keep the molecular geometry fixed, allowing only relative molecular positions to change. This corresponds to a rigid-body optimisation, where only the intermolecular score $Z^{FF}_{inter}$ is calculated. We will refer to this type of optimisation as *Constrained* for the rest of this article.
- Allow the unit cell parameters to change and also allow variations in molecular geometry. In this case, both the $Z^{FF}_{inter}$ and $Z^{KBF}_{intra}$ scores are calculated. We will refer to this type of optimisation as *Full*.

Before each optimisation, hydrogen atom positions were normalised according to neutron diffraction data [27]. The remaining parameters (limiting radius, convergence tolerance, etc.) were left to their default values.

#### Calculation of lattice energies

The lattice energy corresponds to the total stabilisation energy (per molecule) related to the transfer of an isolated gas phase molecule, having no intermolecular interactions, into the crystal, where non-bonded interactions of various nature are set up with surrounding molecules. In the context of force field based methods this energy can be calculated as the sum of interatomic potentials between atoms *i* in a central molecule and atoms *j* in surrounding molecules within a reasonable limiting radius:

$$E_{latt} = \frac{1}{2}\sum _i\sum _j E_{ij}$$

where a factor of one half accounts for each interaction being shared by two molecules. For conformationally flexible molecules, an additional relaxation energy for conformational changes between the stable gas-phase molecule and the molecule in the *in-crystal* conformation should also be included. While the inclusion of this correction has been shown to reduce the overall error in a benchmark study of DFT-D methods against experimental sublimation enthalpies [28], its calculation using intermolecular potentials is not feasible. An alternative is to apply a 2RT thermal correction to the lattice energy, but the effect is considered negligible, if not detrimental, relative to the magnitude of error associated with experimental sublimation enthalpy measurements [9] and general purpose force field methods ($\sim$ 5-15 kJ mol$^{-1}$) [6, 15], and this correction was not applied here. To use Gavezzotti’s words “*no correction is better than a bad correction*”. Thus, here, we compare lattice energies and sublimation enthalpies directly.

Since, as discussed in the previous section, the scores returned by *Crystal Optimiser* do not always correspond exactly to crystal lattice energies, lattice energies were calculated using the CSD-Particle program *VisualHabit* [7]. A comparison of *Crystal Optimiser* scores with *VisualHabit* lattice energies, justifying this decision, can be found in the supporting information.

For each of the systems considered in this work, calculations of *VisualHabit* lattice energies were repeated four times, each with different input crystal structures:

(i) Crystal structure as read from the CSD, no further processing (No opt).
(ii) Crystal structure as read from the CSD, but with hydrogen atom positions normalised according to neutron diffraction data (X-H normalised).
(iii) Crystal structure optimised with constraints in *Crystal Optimiser* (Constrained opt).
(iv) Crystal structure fully optimised with *Crystal Optimiser* (Full opt).

Note that missing hydrogen atoms were added to all structures prior to calculation, when needed. Atomic charges were also redetermined for each input structure before each *VisualHabit* calculation.

In the case of the CLP force field, an additional correction to account for the alignment of molecular dipoles in polar crystal structures was also considered, as described in the documentation for the original program, now available through the MiCMoS suite [29]:

$$E_{dipole} = -1389.355\frac{2\pi }{\mu ^2}(3NV_{cell})$$

where $\mu$ is the dipole moment of the unit cell, *N* is the number of molecules in the unit cell and $V_{cell}$ is the unit cell volume.

This correction was not applied to the remaining force fields.

### Comparison of optimised crystal structures

Optimised crystal structure geometries were evaluated with reference to the corresponding CSD entries using the COMPACK method [30], using clusters of 20 molecules with the default distance and angle tolerances of 20% and $20^{\circ }$, respectively. Additional metrics, namely RMSD values for the unit cell angles and unit cell axes lengths, the relative change of unit cell parameters compared to the original CSD structures, as well as the differences in molecular geometries between constrained and fully optimised structures (atomic coordinates, bond angles and torsion angles) were also calculated, as described in the Supplementary Information.

## Results and discussion

Lattice energies were calculated for datasets using each force field, as described in the methods. For the CG2019 set, we report an overall assessment of the geometry change after optimisation and accuracy of calculated lattice energies. This analysis was also performed for subsets of several classes of compounds. The results are available in the associated Supplementary Data. Calculated lattice energies for some crystal structures in the CG2019 dataset resulted in very large differences compared to experimental sublimation enthalpies. Rather than removing these outliers, we have decided to include them in our analysis. Some of the entries that resulted in large structural distortions after optimisation are also highlighted and discussed. The assessment of force field performance for the polymorphic dataset was focused on relative rather than absolute energies to aid the comparison with literature data.

### CG2019 dataset

#### Crystal structure optimisation

The COMPACK tool was used to evaluate the structural change introduced by the geometry minimisation by comparing clusters of 20 molecules. COMPACK returns the number of matching molecules, as well as a RMSD value for the structures’ overlap. Ideally, structural optimisation should only introduce small variations to the crystal packing, and the optimised structure should remain representative of the same crystal phase as the corresponding experimental CSD entry, meaning that the number of matched molecules ($N_{match}$) between the compared clusters should be maximal (a match of 20 molecules, in our case) [31, 32]. Table 1 reports the overall results for this comparison, while histograms showing the distributions of these values are available in the Supplementary Information.

**Table 1:** Average values for optimisation results showing the number of matching molecules compared to the original CSD entry (out of a cluster of 20 molecules), $N_{match}$, the associated RMSD-20, the percentage of structures with $N_{match}$ less than 20, the average number of optimisation steps and the average optimisation time

Force field	N	Optimisation type	N$_{match}$	N$_{match}$	RMSD-20	N$_{steps}$	$t_{opt}$
			(out of 20)	(% less than 20)	(Å)		(s)
CLP	663	Constrained	19.7	3.62	0.17	8.6	40.9
		Full	19.8	3.02	0.29	21.5	88.6
UNI	664	Constrained	19.8	1.81	0.12	8.0	14.5
		Full	19.7	4.22	0.21	21.6	34.4
CSD-OPCS16	664	Constrained	19.8	1.95	0.11	8.0	15.2
		Full	19.7	3.45	0.17	16.0	26.2
DreidingII	664	Constrained	19.8	2.41	0.15	10.5	549.5
		Full	19.6	5.12	0.24	18.4	2085.6
Momany	550	Constrained	19.7	4.00	0.16	10.2	362.0
		Full	19.5	7.45	0.24	22.7	1704.2

The average $N_{match}$ is slightly below 20 for all force fields, which indicates that in most cases structural optimisation results in the same crystal phase as the starting crystal structure. As expected, the average RMSD-20 values increase on changing from a constrained to a full optimisation, as more degrees of freedom become available. The proportion of crystal structures with $N_{match}$ less than 20 also increases in changing the optimisation type, except for the CLP force field, for which full optimisation results in a higher number of matching structures. We note that relaxing the angle and distance tolerances used for the COMPACK comparison would likely reduce the number of mismatched structures and that we have decided to use the default values here to aid the identification of possible outliers. Looking in more detail, the percentage of crystal structures with a low packing similarity compared to the original CSD entries are in the range of 1.81% to 4% for the constrained optimisations and in the range of 3.02% to 7.45% for the full optimisations. In both cases, the structures optimised with the Momany force field show the largest relative variation.

As for the average changes in unit cell parameters for full optimisations (reported in Supplementary Table 4), where unit cells are allowed to vary within a specified volume change limit (10%, by default), results are similar for all force fields. The average RMSD values for the unit cell axes are around 0.3 Å, while, on average, unit cell angles vary by less than 2 degrees. As for the relative changes for the unit cell axes, often reported in similar studies, they are also similar across all force fields, with average relative changes for the a, b and c axes around 4%, 2% and 3%, respectively. The CLP-optimised structures have unit cells that are slightly more distorted compared to structures optimised with the other force fields.

Table 1 also reports the average number of optimisation steps ($N_{steps}$) required to reach convergence, as well as the average optimisation time ($t_{opt}$). As expected, both $N_{steps}$ and $t_{opt}$ increase in case of a full optimisation. The time required for the calculations is noticeably less for the CLP, UNI and CSD-OPCS16 force fields, for which an automatic differentiation algorithm is used in *Crystal Optimiser*. For these force fields, the time for an optimisation scales roughly linearly with the number of atoms in the structure, while this scaling is roughly quadratic in the case of the DreidingII and Momany force fields (see plots in the Supplementary Information).

**Fig. 1:**
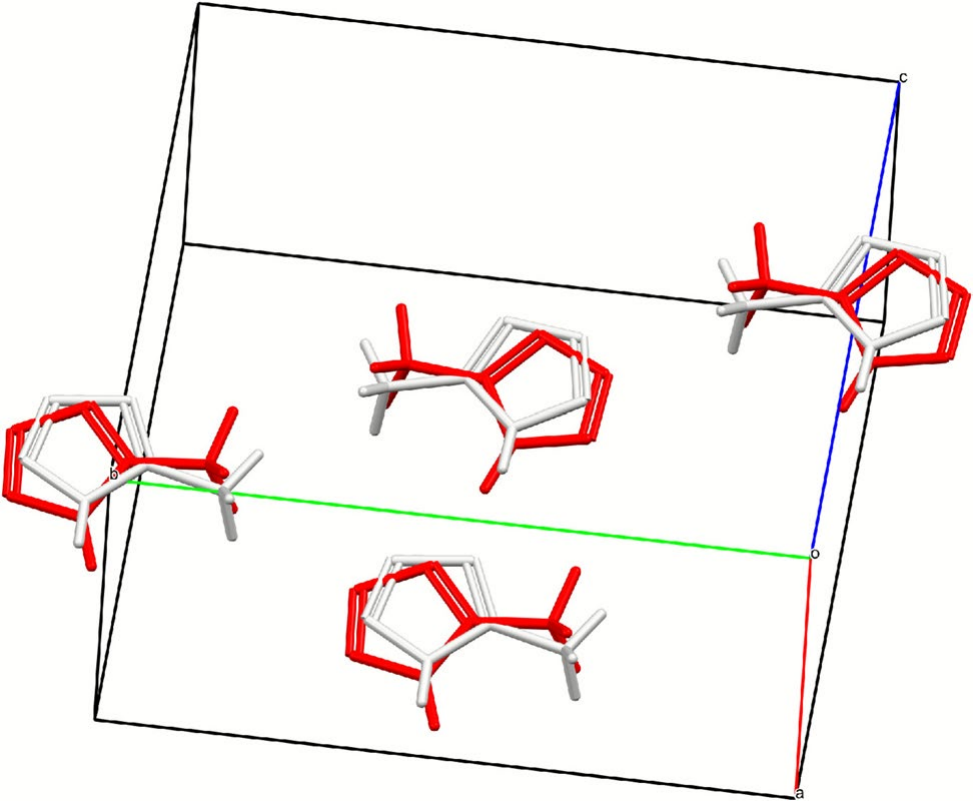
Crystal packing overlay of unoptimised (white) and optimised (red) crystal structures of 5-Methyl-1H-tetrazole (FIZZOD). The structure optimised with constraints using DreidingII is shown here

A closer analysis of the entries that have a low $N_{match}$ shows that there are structures for which optimisation with multiple, if not with all of the available force fields can result in significant structural distortion (at least within the tolerances used for our comparison). For example, constrained optimisations of 5-Methyl-1H-tetrazole (CSD refcode FIZZOD) with all force fields yield structures in which the tetrazole rings are slightly rotated (Fig. 1). Picric acid (CSD refcode PICRAC) and 2,3-dinitrotoluene (CSD refcode FIHLOY) also result in a mismatch of crystal packing if fully optimised with any of the force fields available.

**Fig. 2:**
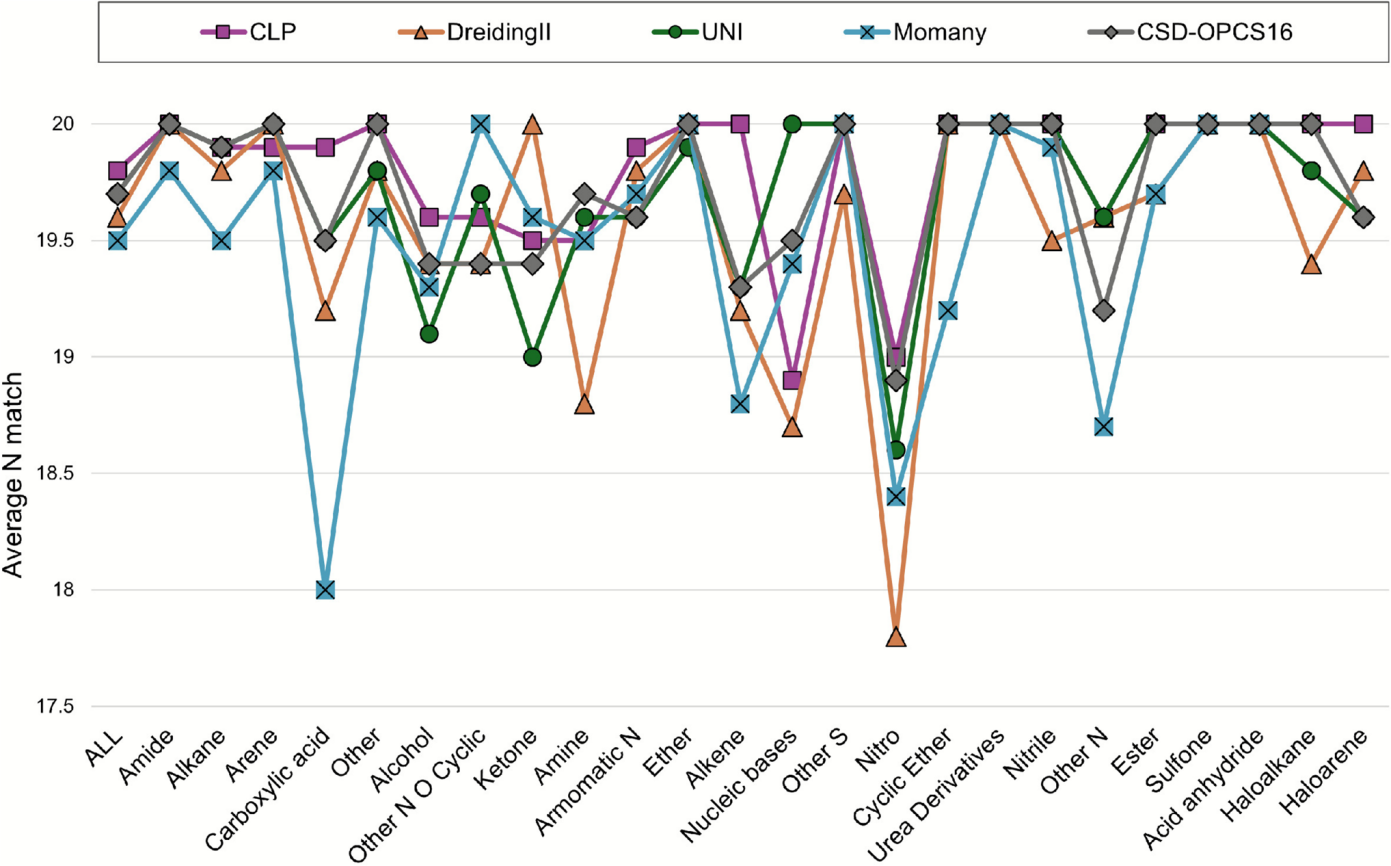
Average number of matching molecules for clusters of 20 molecules as calculated with COMPACK; values are for comparisons of fully-optimised structures with original CSD entries

Figure 2 shows the average $N_{match}$ values for different compound classes for fully optimised structures. Structures containing nitro groups consistently result in lower similarity scores for all force fields. In the case of the Momany force field, structures containing carboxylic acid groups, where optimising molecular geometries results in the rotation of these groups, also have a lower average similarity compared to the original CSD entries.

**Fig. 3:**
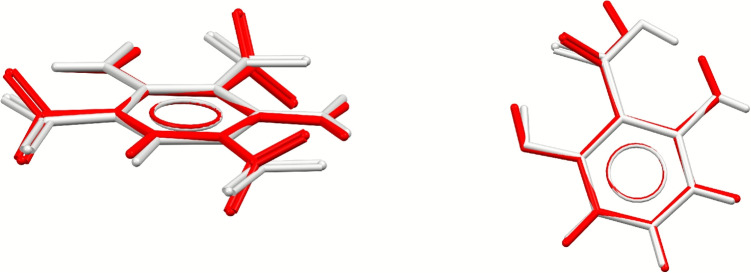
Overlay of unoptimised (white) and optimised (red) molecules for DATNBZ01 (left) and LEZJAB01 (right), showing the rotation of nitro groups and the loss of intramolecular hydrogen bonds, respectively, when the molecular geometry is optimised

To evaluate the extent of changes in molecular geometries during full optimisations, we have also calculated descriptors to compare atomic positions (molecular overlay), bond angles and torsions, as described in the Supplementary Information, where histograms showing the distributions of these values are also reported. Results are consistent across all force fields, and show that variations in molecular geometries are quite small in most cases, as could be expected due to the optimised molecules having to satisfy requirements imposed by the crystal packing. The majority of structures have RMSD values for overlaid atomic positions that are less than 0.1 Å. Changes in bond angles are also small and follow normal distributions centred around 0.003$^{\circ }$ and 0.004$^{\circ }$. Finally, most torsion angles change by less than 10$^{\circ }$, and the largest variations (as high as 40$^{\circ }$) are typically associated with -NO${_2}$ groups, like in the case of 1,3-diamine-2,4,6-trinitrobenzene (DATNBZ01, Fig. 3 left), where this rotation results in a low COMPACK similarity score. We also observed considerable changes in molecular geometries in some structures containing intramolecular hydrogen bonds, 2,6-dihydroxybenzoic acid (LEZJAB01, Fig. 3 right) being a notable case. For this compound, optimisation of the molecular geometry results in the rotation of two hydroxyl groups and in the loss of the intramolecular bond, resulting in overall loss of accuracy in calculated lattice energy. Chickos and Gavezzotti have suggested that some intramolecular hydrogen bonds are likely to be retained during sublimation [6]. Thus, avoiding optimisation of molecular geometries might be preferable in these cases. As a general observation, we found that larger changes in crystal packing during optimisation are found for small rigid molecules containing rings, especially N-heteroaromatic rings, in the case of constrained optimisations, and for molecules containing nitro groups in the case of full optimisations.

#### Comparison of calculated lattice energies with experimental sublimation enthalpies

Lattice energies for the compounds in the CG2019 dataset have already, of course, been calculated by Professor Gavezzotti using the original CLP code. Thus, it seems reasonable to first compare our results to the original ones. Lattice energies reported in ref. [6] were calculated for unoptimised crystal structures from the CSD, after normalisation of hydrogen atom positions (“Calculation of lattice energies” section, structure input (ii)). The corresponding lattice energies calculated for this work show good agreement, although not perfect, with the original ones, with a mean absolute error of less than 2 kJ mol$^{-1}$. An analysis of the main outliers (39 structures with $\Delta E_{latt}>$5 kJ mol$^{-1}$ and 9 structures with $\Delta E_{latt}>$10 kJ mol$^{-1}$) showed that discrepancies between our implementation and the original CLP are due to small differences in atom types assignment, especially for structures containing *aromatic core* C atoms. In some cases, these differences result in less accurate calculated sublimation energies, like for benzotriazole (BZTRAZ01), where the difference with the experimental value is 12 kJ mol$^{-1}$ for the original CLP and 51 kJ mol$^{-1}$ for our implementation. In other cases, however, the reverse is true. Like for methyl carbamate (FEPGEM) for example, where the difference with the experimental value is 10.5 kJ mol$^{-1}$ for the original CLP and 0.6 kJ mol$^{-1}$ for our implementation.

**Table 2:** Percentage of entries with a difference of calculated vs. experimental lattice energy (%D) less than a threshold value from ref. [6] and from this work, together with the associated RMSD in kJ mol$^{-1}$

	CLP 2019 publication		CLP this work	
%D less than	% (out of 669)	RMSD	% (out of 663)	RMSD
5	31	2.7	30.8	3.2
10	59	6.2	56.6	6.0
15	74	8.3	71.9	8.2
20	82	9.6	82.1	9.9
25	90	11.6	88.2	11.4
30	93	16.7$^a$	92.8	12.9

$^{a}$12.5 kJ mol$^{-1}$ recalculated from data in ref. [6]

Table 2 shows the result for the comparison of lattice energies from the two methods with sublimation enthalpies. The table reports the percentage of the calculated structures that have a relative error less than a certain threshold (%D), together with the associated RMSDs in kJ mol$^{-1}$. Following the description provided by Gavezzotti, the results shown in the table can be interpreted as the chance of matching the experimental sublimation enthalpy within a certain accuracy. Despite the discrepancies described previously, there is a good agreement between our results and those presented in the referenced paper. Of particular note is the lower RMSD value for the structures with an absolute difference less than 30%, for which we get an RMSD of 12.9 kJ mol$^{-1}$ compared to 16.7 kJ mol$^{-1}$. We have found this to be an error in the original publication, likely unintentional. Recalculating this value from the original data, we get an RMSD of 12.5 kJ mol$^{-1}$, much closer to our results. We have calculated similar tables for the remaining data series presented in this work (i.e., various types of input crystal structures and different force fields), and they can be found in the associated Supplementary Data.

Table 3, instead, reports descriptors (mean absolute error, root mean square deviation, maximum absolute error and average percentage error) for our calculations, as compared to sublimation enthalpy data from the CG2019 dataset.

**Fig. 4:**
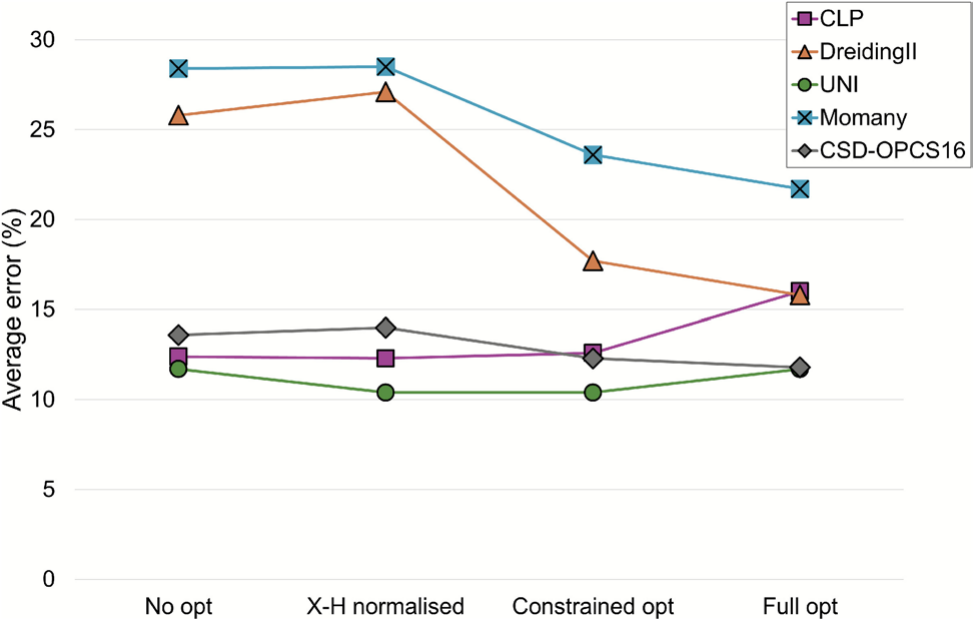
The average percentage error (%) for lattice energies calculated with CLP (squares), UNI (circles), CSD-OPCS16 (diamonds), DreidingII (triangles) and Momany (crossed squares) compared to sublimation energies from the CG2019 dataset

Regarding the overall results, the DreidingII and Momany force fields have the lowest accuracy, with average errors larger than 25% in the case of unoptimised structures (Fig. 4). For DreidingII, this is likely because it was parameterised for a wide range of molecular simulations, not specifically for crystal structures [22]. The Momany potential, instead, was parameterised using experimental crystal structures of hydrocarbons, carboxylic acids, amines, and amides [23], but the CG2019 dataset used here consists of more varied classes of compounds. Force fields that were parameterised using crystal structures that more closely resemble the ones of the CG2019 set - CLP, UNI and CSD-OPCS16 - conversely, have a higher accuracy, with error of less than 15% in most cases.

**Table 3:** Mean absolute error (MAE), RMSD, average percentage error (AVG. ERR.), and absolute maximum error (MAX ERR.) of calculated lattice energies versus experimental sublimation enthalpies for the CG2019 dataset. All units are kJ mol$^{-1}$ except for the percentage error. Descriptors recalculated from the data in ref. [6] are also reported

Force field	Calculation type	MAE	RMSD	AVG. ERR. (%)	MAX ERR.
CLP$^a$	No opt	11.8	16.4	12.4	96.6
	X-H normalised	11.9	16.8	12.3	102.5
	X-H normalised (2019 results)	11.7	16.8	12.1	102.6
	Constrained opt	12.1	16.5	12.6	103.6
	Full opt	15.4	20.2	16.0	113.0
UNI	No opt	11.4	16.4	11.6	99.6
	X-H normalised	10.2	14.3	10.4	72.4
	Constrained opt	10.2	14.3	10.4	74.0
	Full opt	11.6	16.1	11.7	82.8
CSD-OPCS16	No opt	13.9	19.3	13.6	116.1
	X-H normalised	14.2	18.2	14.0	82.6
	Constrained opt	12.6	16.3	12.3	80.2
	Full opt	12.0	15.6	11.8	75.3
DreidingII	No opt	26.9	58.0	25.8	703.0
	X-H normalised	28.5	39.9	27.1	181.5
	Constrained opt	18.2	26.1	17.7	253.8
	Full opt	15.9	24.3	15.8	332.1
Momany	No opt	29.8	50.6	28.3	502.8
	X-H normalised	29.7	40.4	28.5	256.8
	Constrained opt	24.6	32.8	23.6	127.7
	Full opt	22.5	30.8	21.7	127.2

$^{a}$A correction for unit cell dipole has been added to CLP energies

Focusing on the individual results for each force field, we can see that optimising crystal structures before performing the lattice energy calculations results in an increase in accuracy for all force fields, with the exception of CLP and UNI. This is particularly evident for the DreidingII force field, where the average percent error is decreased from 25.8% to 15.8%. The repulsive term of the DreidingII potential is particularly sensitive to the position of hydrogen atoms in hydrogen-bonding groups, for example when a carboxylic acid dimer is formed between two molecules (see next section). Optimising the crystal structure, in these cases, helps to increase stability by reducing this repulsive term. As for CLP and UNI, the decrease in accuracy after optimisation can be explained, once again, with the fact that these force fields were parameterised using unoptimised crystal structures [13, 19, 20] and therefore optimisation does not necessarily result in better estimates of sublimation enthalpies. For CLP, this effect is more pronounced, likely because this force field contains terms that show a stronger dependence on interatomic distance, particularly the coulombic term (r$^{-1}$) and, secondarily, the polarisation term (r$^{-4}$). UNI, instead, consists of only one attractive and one repulsive term, both of which have a relatively weak dependence on interatomic distances. This difference is reflected in the larger decrease in accuracy for CLP (from 12.3% to 16% error) compared to UNI (from 10.4% to 11.7% error), when optimisation is introduced.

Sixty-nine of the compounds from the CG2019 dataset crystallise in a polar space group. For these crystal structures, an additional contribution to the lattice energy due to the alignment of strong molecular dipoles should also be included, as described in “Calculation of lattice energies” section. For most crystal structures, this correction is expected to be quite small [13], and here we have only applied it to the results for the CLP force field. This was done mostly to maintain consistency with the original CLP implementation, and we have found that not including this correction had only marginal effects on the overall results (the average percentage error for X-H normalised CLP calculations increases from 12.3% to 12.4%). For the 69 polar crystals in our dataset, this correction averaged to -4.6 kJ mol$^{-1}$, with 2-methyl-2-nitro-1,3-propanediol (MENPDL) being a notable case with a contribution due to cell dipoles as high as -48 kJ mol$^{-1}$.

Our results show that, if one desires to use the DreidingII or Momany force fields for their calculations, they should consider the option of optimising their crystal structure to calculate more accurate lattice energies. The CLP, UNI and CSD-OPCS16 force fields are more forgiving and, despite some decrease in accuracy after optimisation, remain a versatile option for many types of calculations, with a higher overall accuracy compared to DreidingII and Momany. Additionally, the possibility of applying a correction to energies in the case of polar crystal structures should also be considered, although in most cases its effect will be quite small.

#### Results by compound class

**Fig. 5:**
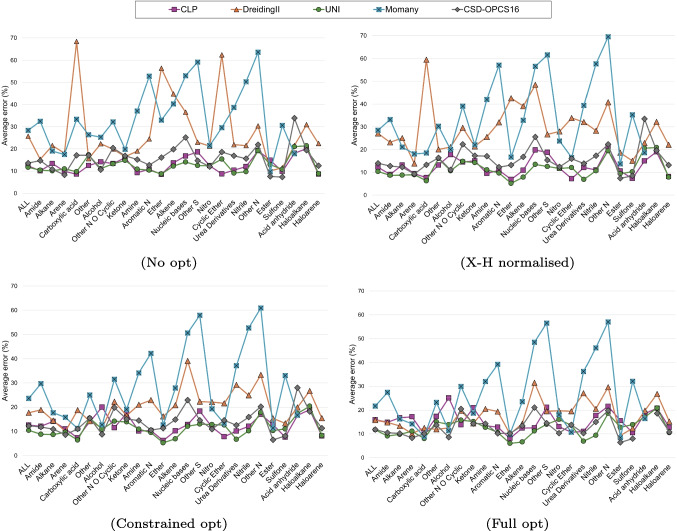
Average percentage error for calculated lattice energies compared to experimental sublimation enthalpies analysed for 24 classes of compounds. Results are presented for unoptimised structures, without (No opt) and with (X-H normalised) hydrogen atom normalisation, and for optimised structures with (Constrained opt) and without (Full opt) constraints

Figure 5 shows distributions of the average percentage error for calculated lattice energies (vs. sublimation enthalpies) for 24 classes of compounds, assigned as described in “Classification of types of compounds” section. Tables 7-11 in the Supplementary Information report the corresponding values.

From the plots, we can see that CLP, UNI and CSD-OPCS16 show less variation across different types of compounds, whereas DreidingII and Momany show rather large errors in some cases, but errors comparable to the other force fields in others. In the case of esters, for example, the average error of DreidingII for unoptimised structures is only 10.1% and that of Momany is 9.7%, compared to 14.9% for CLP and 12.9% for UNI. Only CSD-OPCS16 has a lower error in this case (7.6%).

As already discussed, optimising a crystal structure can improve the energies calculated with DreidingII or Momany. This is particularly evident for carboxylic acids, where the average error is reduced from 68.4% to only 12.5% for DreidingII and from 33.4% to 8.9%. Similarly, for ethers, the error is reduced from 56.4% to 9.9% in the case of DreidingII and from 33% to 9.9% in the case of Momany. For this last force field, some classes of compounds (for example aromatic N-heterocyclic rings and nitriles) don’t benefit from optimisation, and the errors remain rather large.

Overall, we have seen that the CLP and UNI lattice energies for optimised structures are less accurate than for the unoptimised ones. There are a few classes of compounds, however, for which constrained optimisation does result in a reduction of the average error, like in the instance of carboxylic acids, alkenes and urea derivatives.

It is difficult to provide a definitive evaluation of the *best-performing* force field, and our results show that the accuracy of calculated lattice energies (when compared to sublimation enthalpies) will vary depending on the nature of the compound of interest, as well as the strategy chosen for the calculation. The force fields presented in this work have both advantages and disadvantages, and the suitability of each force field to the system of interest will need to be evaluated from case to case.

### Polymorphic dataset: relative lattice energies from quantum mechanical methods versus force fields

After looking at the CG2019 dataset, we turn our attention towards evaluating the applicability of the CLP, UNI, DreidingII and Momany force fields to the issue of ranking polymorphs of compounds of interest. CSD-OPCS16, having results comparable to UNI, was not included in this analysis.

**Fig. 6:**
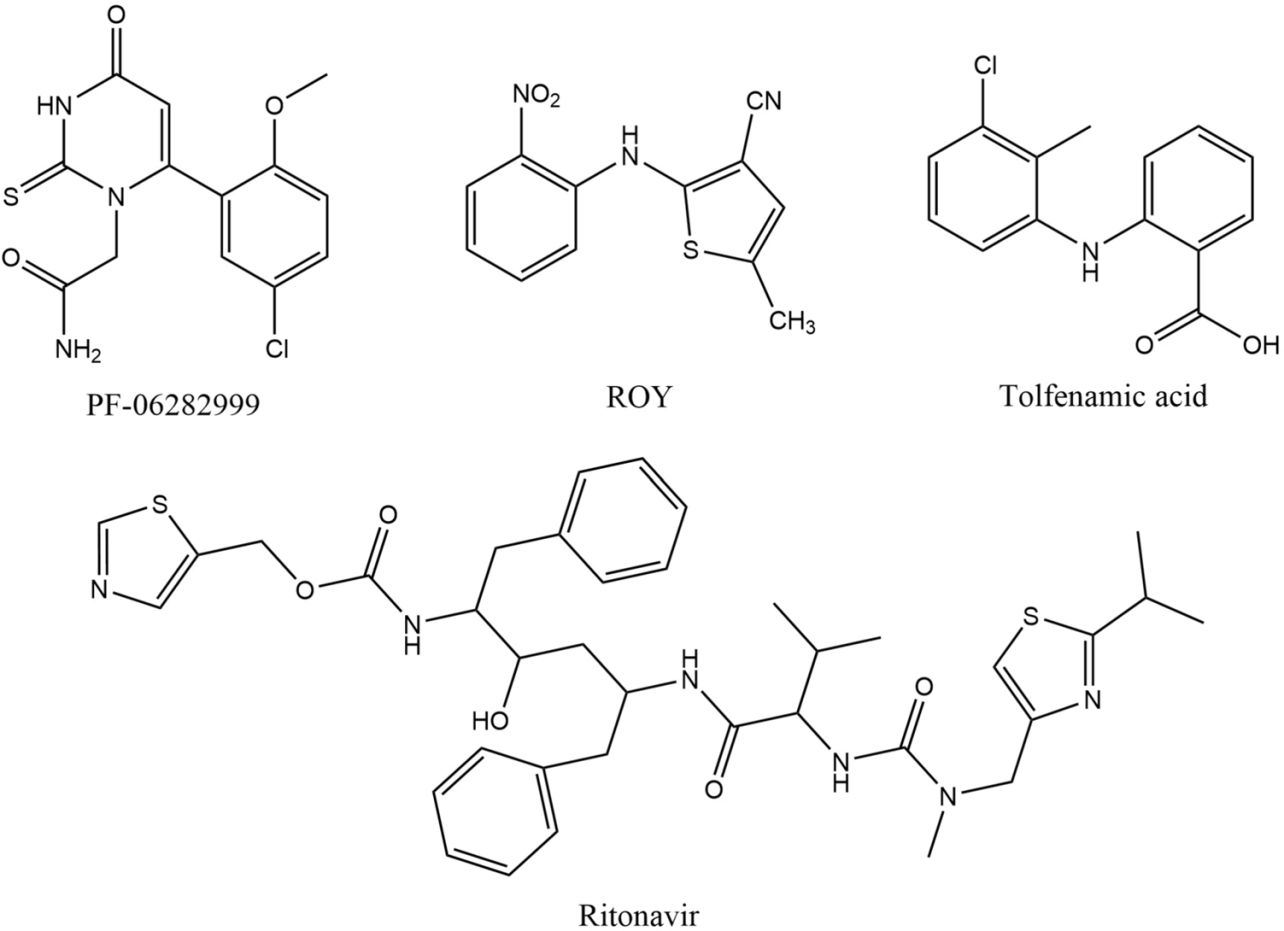
Chemical diagrams of the four polymorphic systems studied: PF-06282999, ROY, tolfenamic acid, and ritonavir

Here, we are curious to evaluate how well these parameter-based methods can compare against state-of-the-art quantum mechanical (QM) methods that are now routinely applied to the refinement of crystal energy landscapes resulting from large-scale CSP studies. The compounds with molecular structures reported in Fig. 6 were chosen due to their renown and, most importantly, because pre-existing calculation results could easily be accessed.

Lattice energies used for this discussion were calculated using fully-optimised structures, which were compared to the respective CSD entries with COMPACK to ensure that their crystal packing was representative of the polymorph of interest. For the two systems containing chlorine atoms (tolfenamic acid and PF-06282999), energies were not calculated with Momany, since this force field has no parameters for halogen atoms.

#### Ritonavir

The relative energies of forms I and II of ritonavir have been calculated previously at the PBE-GD2 and PBE-TS levels of theory using periodic DFT [33, 34]. Both methods found the difference for the intermolecular component of the lattice energy between the stable form II and the metastable form I to be either 39.1 or 34.4 kJ mol$^{-1}$. All of the considered force fields agree in identifying form II as the stable polymorph, with an energy difference in the same order of magnitude as that found with DFT methods, as shown in Table 4.

**Table 4:** Relative lattice energies (kJ mol$^{-1}$) calculated at PBE-GD2 and PBE-TS levels of theory (literature values) and using the CLP, DreidingII, UNI, and Momany force fields after a full optimisation

Polymorph	CSD refcode	PBE-GD2	PBE-TS	CLP	DreidingII	UNI	Momany
I	YIGPIO02	39.10	34.40	21.52	23.71	20.70	36.61
II	YIGPIO03	0.00	0.00	0.00	0.00	0.00	0.00

#### ROY

**Fig. 7:**
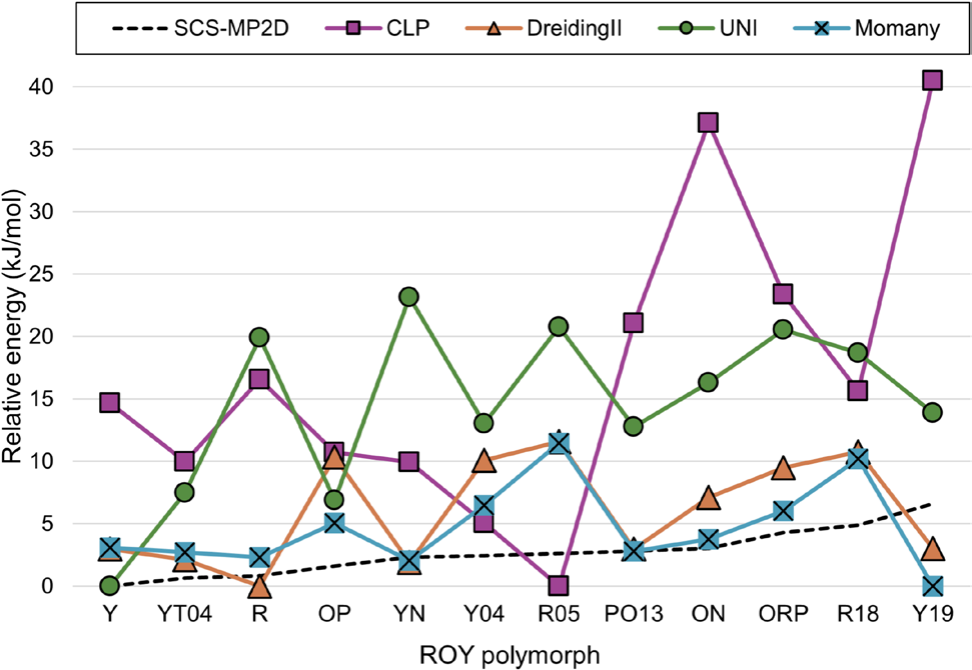
Comparison of relative lattice energies of ROY polymorphs calculated by force fields versus SCS-MP2D

**Fig. 8:**
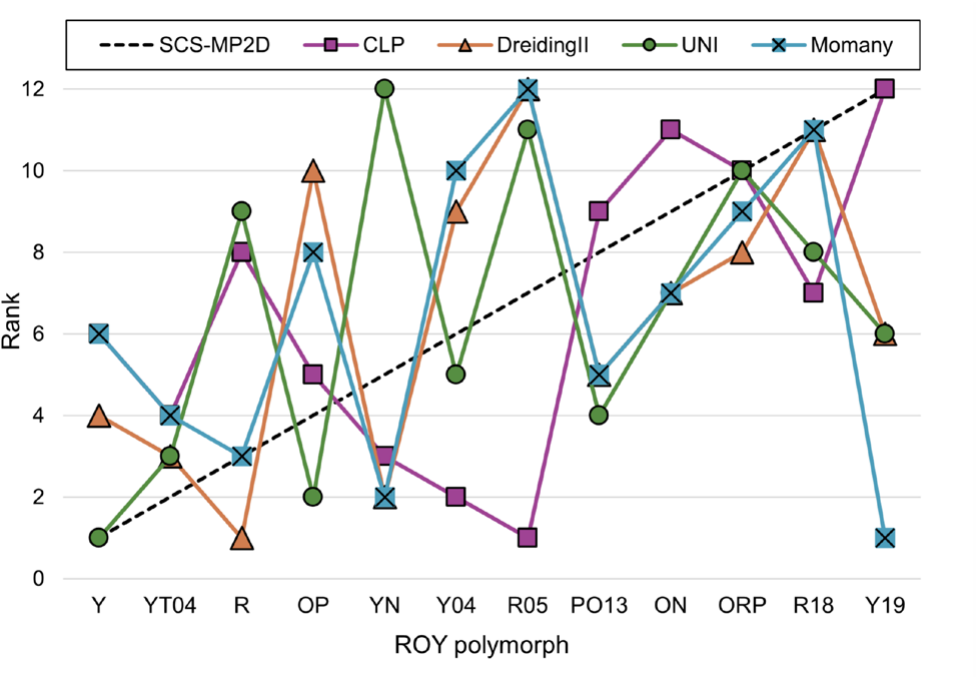
Comparison of rank order of ROY polymorphs calculated by force fields versus SCS-MP2D

Lattice energies of 12 polymorphs of ROY (Y, YT04, R, OP, YN, Y04,R05, PO13, ON, ORP, R18 and Y19) have previously been calculated at the SCS-MP2D level of theory as part of a CSP study [35]. Polymorphs of this compound have very small energy differences according to DFT results. Considering the larger uncertainties associated with force field energies, relative energies calculated using the Momany and DreidingII force fields (Table 5) are in reasonable agreement with those from SCS-MP2D calculations, while this is not the case for energies calculated with UNI and CLP. Plots of relative energies (Fig. 7) and ranks (Fig. 8) indicate no clear correlation with results of SCS-MP2D calculations, and none of the force fields finds the same energy ranking. Interestingly, each force field seems to find a different stable polymorph for ROY: UNI correctly identifies polymorph Y; DreidingII finds polymorph R; Momany finds polymorph Y19; finally, CLP finds R05 as the most stable polymorph. For the latter, which crystallises in a polar space group, the contribution to the CLP lattice energy due to the unit cell dipole is quite large (-60 kJ mol$^{-1}$) and strongly contributes to its stabilisation, while the other polymorphs, which are centrosymmetric and lack this correction, have considerably higher energies (at least +5 kJ mol$^{-1}$). This is also the case for energies calculated with UNI, which, despite having been found to be the most accurate when reproducing sublimation enthalpies for the CG2019 dataset, also fails to account for the very small energy differences identified by DFT. Here, the best results in terms of absolute relative energies are obtained for the Momany force field, which is representative of the fact that the choice of the right force field is contingent on the type of compound for which energies are to be calculated.

**Table 5:** Ranks and corresponding relative energies (kJ mol$^{-1}$) for ROY polymorphs calculated at the SCS-MP2D level of theory (literature values)

Polymorph	Rank					Relative energy				
	SCS-MP2D	CLP	DreidingII	UNI	Momany	SCS-MP2D	CLP	DreidingII	UNI	Momany
Y	1	6	4	1	6	0	14.7	3.0	0.0	3.1
YT04	2	4	3	3	4	0.64	10.0	2.1	7.5	2.7
R	3	8	1	9	3	0.83	16.6	0.0	19.9	2.3
OP	4	5	10	2	8	1.61	10.7	10.3	6.9	5.1
YN	5	3	2	12	2	2.31	9.9	1.9	23.1	2.0
Y04	6	2	9	5	10	2.42	5.0	10.1	13.0	6.5
R05	7	1	12	11	12	2.61	0.0	11.6	20.8	11.5
PO13	8	9	5	4	5	2.8	21.1	3.0	12.8	2.8
ON	9	11	7	7	7	3.02	37.1	7.1	16.3	3.7
ORP	10	10	8	10	9	4.29	23.4	9.5	20.5	6.0
R18	11	7	11	8	11	4.86	15.6	10.7	18.7	10.2
Y19	12	12	6	6	1	6.61	40.5	3.0	13.9	0.0

#### Tolfenamic acid

Relative energies of polymorphs I - IX of tolfenamic acid (TFA) have been previously calculated at the PBE-MBD level of theory using periodic DFT [36].

**Fig. 9:**
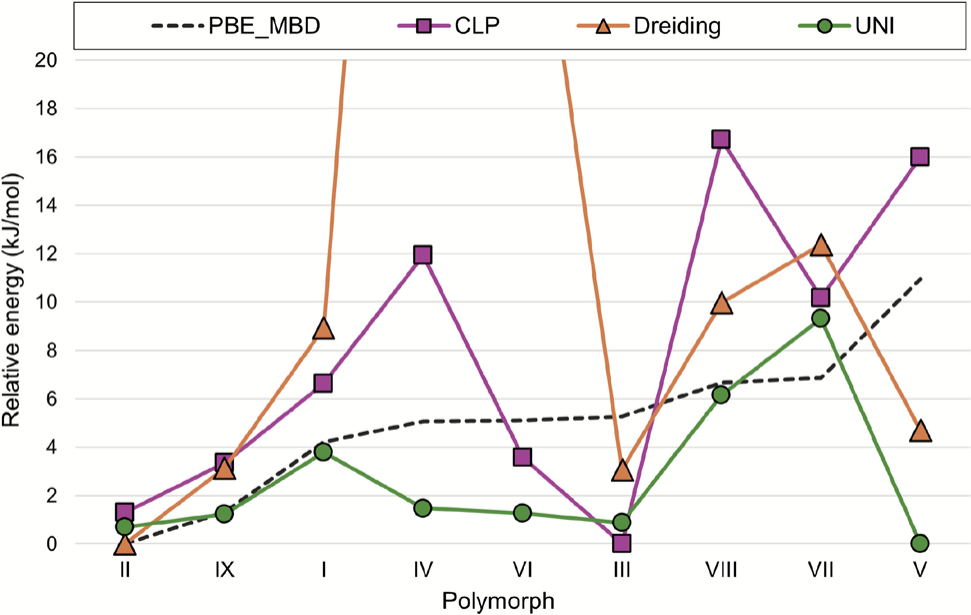
Relative lattice energies of TFA polymorphs calculated by force fields versus PBE-MBD

**Fig. 10:**
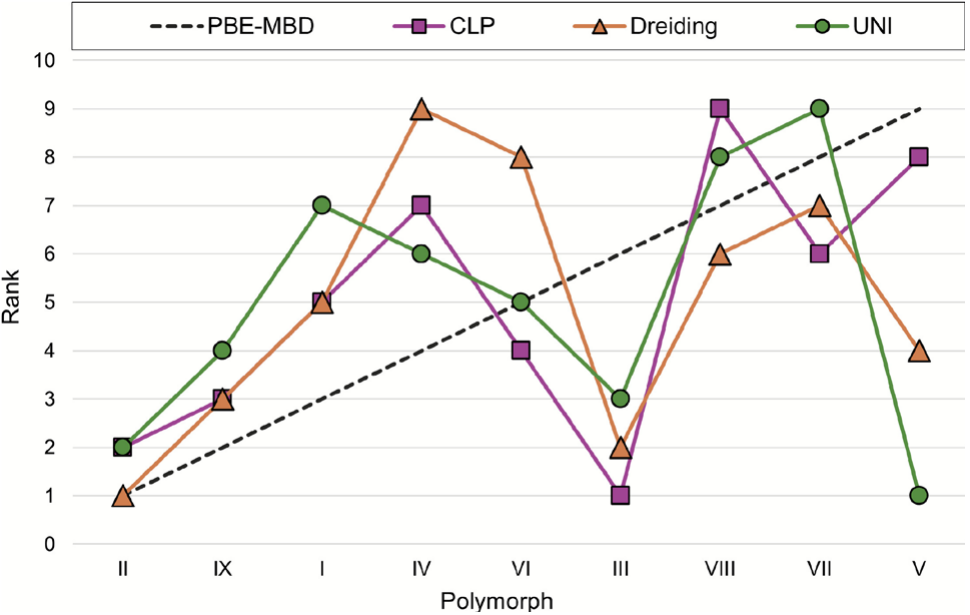
Rank order of TFA polymorphs calculated by force fields versus PBE-MBD

Comparison with those calculated with CLP, DreidingII, and UNI (see Figs. 9 and 10) shows that form II is identified as the most stable polymorph only in the case of DreidingII, while both CLP and UNI rank it as the second lowest form, with small energy differences of +1.3 and +0.7 kJ mol$^{-1}$, respectively. All considered, relative energies calculated with force fields have magnitude comparable to DFT ones (and smaller than the expected uncertainty), with the evident exception of forms IV and VI as ranked with DreidingII, which have relative energies of +62.1 and +31.9 kJ mol$^{-1}$, respectively. In the case of UNI, relative energy differences are actually smaller than DFT ones in most cases. However, using this force field, form V is identified as the most stable polymorph, while according to DFT it is in fact the least stable one. CLP does indeed identify this form (V) as one of the least stable, although it also identifies form III as the stable polymorph. It is worth noting, though, that analysis of the CG2019 dataset (“Comparison of calculated lattice energies with experimental sublimation enthalpies” section) showed that this force field is better suited for unoptimised structures, and that CLP does indeed identify form II as the stable polymorph when the lattice energy for the unoptimised CSD entry is calculated instead. Curiously, also UNI and DreidingII identify form III as one of the most stable forms.

#### PF-06282999

Four polymorphs of PF-06282999 have previously been extensively evaluated using both informatics and energetics methods, where relative lattice energies calculated at the PBE-TS and optPBE-vdW levels of theory were reported [37]. Both DFT results agree closely; Forms 1-3 have similar stabilities (within 2.5 kJ mol$^{-1}$), form 3 is most stable, while form 4 is significantly less stable (+10-12 kJ mol$^{-1}$).

**Fig. 11:**
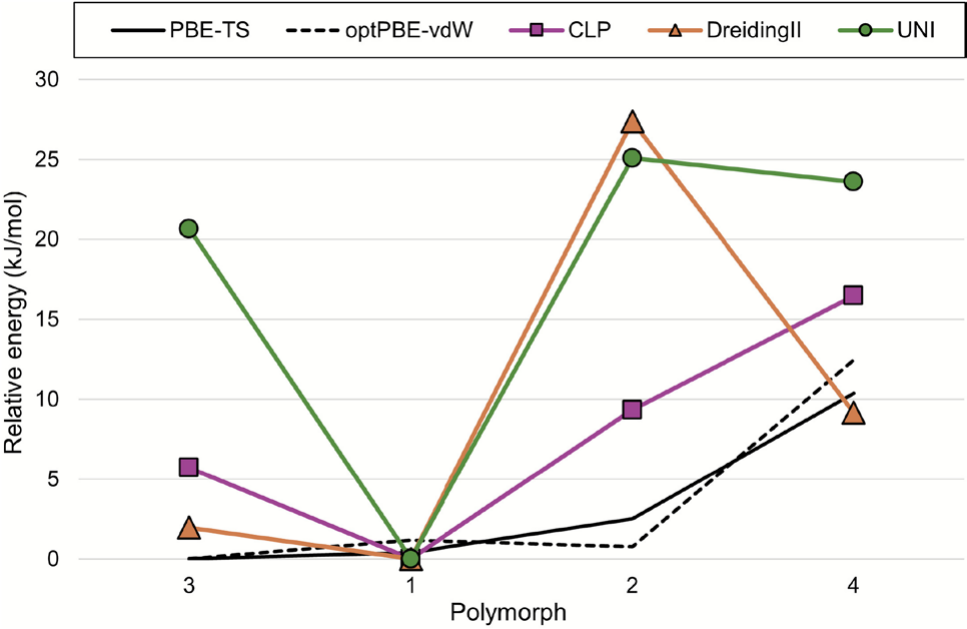
Comparison of relative energies of PF-06282999 polymorphs calculated by each force field versus those calculated at PBE-TS and optPBE-vdW levels of theory from the literature

All force fields consistently calculated polymorph 1 as the lowest in energy (Fig. 11). CLP ranked form 4 as least stable (+16.5 kJ mol$^{-1}$), in agreement with DFT, while UNI and DreidingII rank form 2 the least stable (+25.1 and +27.4 kJ mol$^{-1}$, respectively).

Overall, CLP agrees with DFT within our expected error (15.4 kJ mol$^{-1}$ mean absolute error for full optimisations), while UNI and DreidingII do not. The previous energetics study, which investigated gas phase conformations, showed that forms 2 and 3 contain molecules with higher geometric strain in the crystal than in forms 1 and 4; the intramolecular energetic contribution is larger in forms 2 and 3. Since the force fields applied in this study do not account for the intramolecular contribution to the overall energy, the deviations from DFT relative energies for forms 2 and 3 are likely due to neglecting the intricate interplay between inter- and intra-molecular interactions towards overall stability. This can be rationalised by recent findings that up to 40% of intermolecular stabilisation energy can compensate for intramolecular contributions, Chattopadhyay et al. [38] so general purpose force fields – which do not properly account for intramolecular energetics – should be used with caution when dealing with flexible molecules such as PF-06282999.

## Conclusions

In this work, we have evaluated the performance of five force fields available in the CSD Software (CLP, UNI, CSD-OPCS16, DreidingII and Momany) against the prediction of sublimation enthalpies for a large dataset and towards reproducing relative stabilities and rankings for families of polymorphs. We have found that these force fields can predict experimental sublimation enthalpies within the expected accuracy, and that results can sometimes be improved if crystal structures are optimised. Overall, the UNI force field showed the lowest degree of error, followed by CLP and CSD-OPCS16. DreidingII and Momany were found to have the largest overall errors, although these were considerably reduced for optimised crystal structures. It is important to note that the accuracy of each force field is strongly dependent on the chemistry of the system of interest, and that, for some classes of compounds, even DreidingII and Momany have smaller errors compared to the other force fields.

This work also provided an opportunity to perform a large scale evaluation of the geometry of crystal structures optimised with the newly introduced *Crystal Optimiser*. Our analysis showed that structural optimisation results in notable changes of molecular packing in about 4% of cases, while changes in unit cell parameters remain small and comparable to those found for structures optimised with more expensive DFT methods. The largest structural changes were found for crystal structures of compounds containing nitro groups and intramolecular hydrogen bonds.

As for the performance of these force fields when ranking families of polymorphs, we have found that the small energy differences between polymorphs typically found experimentally and from DFT calculations are poorly reproduced by force fields, which have a higher intrinsic uncertainty. This is likely due to multiple factors, but mainly to the inability of these methods to account for important conformational changes between molecules in the gas and in condensed phases. In the case of large energy differences, however, calculations with force fields might still prove accurate enough to distinguish between stable and metastable polymorphs, as was shown here for ritonavir.

The analysis provided in this work allows us to draw some general indications for those interested in using force fields available in the CSD Software:

- Due to the implementation of a more efficient optimisation algorithm in *Crystal Optimiser*, structure optimisations using the CLP, UNI, and CSD-OPCS16 force fields are significantly more efficient than that with DreidingII and Momany.
- The CLP force field is more accurate when applied to experimental crystal structures, and both CLP and UNI are most suited for workflows —such as morphology prediction— that typically rely on experimental geometries rather than optimised ones.
- The accuracy of Momany and DreidingII force fields significantly improved with structural optimisation.
- While DreidingII and Momany show the highest Mean Absolute Error, accuracy is highly dependent on the chemistry of the compound of interest, and they can perform better than other force fields in some cases.
- General purpose force fields are generally not reliable for calculating relative stabilities of polymorphs (where energy differences between forms are often smaller than the expected uncertainties).

These can only serve as general indications, and the suitability of force field methods will ultimately need to be carefully determined for each individual case. The reduced computational cost and acceptable accuracy, considering the intrinsic errors associated with experimental sublimation enthalpies, mean that these methods are still extremely valuable when performing large scale studies where lattice energies of a considerable number of crystal structures need to be calculated, like in the early stages of crystal structure prediction. Especially for polymorphs that are characterised by large energy differences, ranking of structures using force fields might successfully be applied to reduce the number of structures to consider for more expensive calculations.

Even when applied to the study of polymorphic compounds, where subtle energy differences might not be accurately captured, structural optimisation involving the use of force fields could assist in providing DFT calculations with input crystal structures that are closer to potential energy minima, thus assisting in reducing the overall computational cost. In the future, these methods could also be improved by finding ways to more accurately describe the overall structural stability, for example by including a CSD-derived intramolecular energy correction.

In conclusion, our study provides insight into the capabilities and limitations of the implemented force field methods, and will hopefully serve as guidance for a wide range of structural scientists.

## Supplementary information

Supplementary Information for this work include a document with additional text figures and tables.

## Supplementary Information

Below is the link to the electronic supplementary material.

Supplementary file 1 (1.30 MB)

## Data Availability

The results presented in this contribution can be reproduced by accessing the associated Supplementary Data, consisting in .csv files with results for calculation on the CG2019 dataset, as well as HTML reports with full results for different compound classes.

## References

[1] Beran GJ, Nanda K (2010) Predicting organic crystal lattice energies with chemical accuracy. J Phys Chem Lett 1(24):3480–3487. 10.1021/jz101383z

[2] Carter DJ, Rohl AL (2014) Benchmarking calculated lattice parameters and energies of molecular crystals using van der Waals density functionals. J Chem Theory Comput 10(8):3423–3437. 10.1021/ct500335b 26588311 10.1021/ct500335b

[3] Bernardes CE, Joseph A (2015) Evaluation of the OPLS-AA force field for the study of structural and energetic aspects of molecular organic crystals. J Phys Chem A 119(12):3023–3034. 10.1021/jp512349r 25733134 10.1021/jp512349r

[4] Nyman J, Pundyke OS, Day GM (2016) Accurate force fields and methods for modelling organic molecular crystals at finite temperatures. Phys Chem Chem Phys 18(23):15828–15837. 10.1039/C6CP02261H 27230942 10.1039/c6cp02261h

[5] Hunnisett LM, Francia N, Nyman J, Abraham NS, Aitipamula S, Alkhidir T, Almehairbi M, Anelli A, Anstine DM, Anthony JE, Arnold JE, Bahrami F, Bellucci MA, Beran GJO, Bhardwaj RM, Bianco R, Bis JA, Boese AD, Bramley J, Braun DE, Butler PWV, Cadden J, Carino S, Červinka C, Chan EJ, Chang C, Clarke SM, Coles SJ, Cook CJ, Cooper RI, Darden T, Day GM, Deng W, Dietrich H, DiPasquale A, Dhokale B, van Eijck BP, Elsegood MRJ, Firaha D, Fu W, Fukuzawa K, Galanakis N, Goto H, Greenwell C, Guo R, Harter J, Helfferich J, Hoja J, Hone J, Hong R, Hušák M, Ikabata Y, Isayev O, Ishaque O, Jain V, Jin Y, Jing A, Johnson ER, Jones I, Jose KVJ, Kabova EA, Keates A, Kelly PF, Klimeš J, Kostková V, Li H, Lin X, List A, Liu C, Liu YM, Liu Z, Lončarić I, Lubach JW, Ludík J, Marom N, Matsui H, Mattei A, Mayo RA, Melkumov JW, Mladineo B, Mohamed S, Momenzadeh Abardeh Z, Muddana HS, Nakayama N, Nayal KS, Neumann MA, Nikhar R, Obata S, O’Connor D, Oganov AR, Okuwaki K, Otero-de-la Roza A, Parkin S, Parunov A, Podeszwa R, Price AJA, Price LS, Price SL, Probert MR, Pulido A, Ramteke GR, Rehman AU, Reutzel-Edens SM, Rogal J, Ross MJ, Rumson AF, Sadiq G, Saeed ZM, Salimi A, Sasikumar K, Sekharan S, Shankland K, Shi B, Shi X, Shinohara K, Skillman AG, Song H, Strasser N, van de Streek J, Sugden IJ, Sun G, Szalewicz K, Tan L, Tang K, Tarczynski F, Taylor CR, Tkatchenko A, Tom R, Touš P, Tuckerman ME, Unzueta PA, Utsumi Y, Vogt-Maranto L, Weatherston J, Wilkinson LJ, Willacy RD, Wojtas L, Woollam GR, Yang Y, Yang Z, Yonemochi E, Yue X, Zeng Q, Zhou T, Zhou Y, Zubatyuk R, Cole JC (2024) The seventh blind test of crystal structure prediction: structure ranking methods. Acta Crystallogr B 80(6):548–574. 10.1107/S2052520624008679 10.1107/S2052520624008679 PMC11789160 39418598

[6] Chickos JS, Gavezzotti A (2019) Sublimation enthalpies of organic compounds: a very large database with a match to crystal structure determinations and a comparison with lattice energies. Cryst Growth Des 19(11):6566–6576. 10.1021/acs.cgd.9b01006

[7] Clydesdale G, Roberts K, Docherty R (1996) HABIT95 – a program for predicting the morphology of molecular crystals as a function of the growth environment. J Cryst Growth 166(1):78–83. 10.1016/0022-0248(96)00056-5, crystal Growth 1995

[8] Li J, Tilbury CJ, Kim SH, Doherty MF (2016) A design aid for crystal growth engineering. Prog Mater Sci 82:1–38. 10.1016/j.pmatsci.2016.03.003

[9] Chickos JS, Acree WE Jr (2022) Enthalpies of sublimation of organic and organometallic compounds. 1910–2001. J Phys Chem Ref Data 31(2):537–698. 10.1063/1.1475333

[10] Abramov YA, Li B, Chang C, Zeng Q, Sun G, Gobbo G (2021) Uncertainty distribution of crystal structure prediction. Cryst Growth Des 21(10):5496–5502. 10.1021/acs.cgd.1c00527

[11] Firaha D, Liu YM, van de Streek J, Sasikumar K, Dietrich H, Helfferich J, Aerts L, Braun DE, Broo A, DiPasquale AG, Lee AY, Le Meur S, Nilsson Lill SO, Lunsmann WJ, Mattei A, Muglia P, Putra OD, Raoui M, Reutzel-Edens SM, Rome S, Sheikh AY, Tkatchenko A, Woollam GR, Neumann MA (2023) Predicting crystal form stability under real-world conditions. Nature 623(7986):324–328. 10.1038/s41586-023-06587-3 37938708 10.1038/s41586-023-06587-3 PMC10632141

[12] Della Pia F, Zen A, Alfè D, Michaelides A (2024) How accurate are simulations and experiments for the lattice energies of molecular crystals? Phys Rev Lett 133:046401. 10.1103/PhysRevLett.133.046401 39121404 10.1103/PhysRevLett.133.046401

[13] Gavezzotti A (2011) Efficient computer modeling of organic materials. the atom–atom, Coulomb–London–Pauli (AA-CLP) model for intermolecular electrostatic-polarization, dispersion and repulsion energies. New J Chem 35(7):1360–1368. 10.1039/C0NJ00982B

[14] Sun H, Jin Z, Yang C, Akkermans RLC, Robertson SH, Spenley NA, Miller S, Todd SM (2016) COMPASS II: extended coverage for polymer and drug-like molecule databases. J Mol Model 22(2):47. 10.1007/s00894-016-2909-0 26815034 10.1007/s00894-016-2909-0

[15] Marchese Robinson RL, Geatches D, Morris C, Mackenzie R, Maloney AG, Roberts KJ, Moldovan A, Chow E, Pencheva K, Vatvani DRM (2019) Evaluation of force-field calculations of lattice energies on a large public dataset, assessment of pharmaceutical relevance, and comparison to density functional theory. J Chem Inf Model 59(11):4778–4792. 10.1021/acs.jcim.9b00601 31638394 10.1021/acs.jcim.9b00601

[16] Bruno IJ, Cole JC, Lommerse JP, Rowland RS, Taylor R, Verdonk ML (1997) IsoStar: A library of information about nonbonded interactions. J Comput Aided Mol Des 11(6):525–537. 10.1023/A:1007934413448 9491345 10.1023/a:1007934413448

[17] Gavezzotti A (2013) Equilibrium structure and dynamics of organic crystals by Monte Carlo simulation: critical assessment of force fields and comparison with static packing analysis. New J Chem 37(7):2110–2119. 10.1039/C3NJ00181D

[18] Dunitz J, Gavezzotti A, Rizzato S (2014) “coulombic compression”, a pervasive force in ionic solids. A study of anion stacking in croconate salts. Cryst Growth Des 14(1):357–366. 10.1021/cg401646t

[19] Gavezzotti A (1994) Are crystal structures predictable? Acc Chem Res 27(10):309–314. 10.1021/ar00046a004

[20] Gavezzotti A, Filippini G (1994) Geometry of the intermolecular XH. cntdot.. cntdot.. cntdot. Y (X, Y= N, O) hydrogen bond and the calibration of empirical hydrogen-bond potentials. J Phys Chem 98(18):4831–4837. 10.1021/j100069a010

[21] Cole JC, Groom CR, Read MG, Giangreco I, McCabe P, Reilly AM, Shields GP (2016) Generation of crystal structures using known crystal structures as analogues. Acta Crystallogr B 72(4):530–541. 10.1107/S2052520616006533 10.1107/S2052520616006533 PMC4971547 27484374

[22] Mayo SL, Olafson BD, Goddard WA (1990) DREIDING: a generic force field for molecular simulations. J Phys Chem 94(26):8897–8909. 10.1021/j100389a010

[23] Momany F, Carruthers L, McGuire RT, Scheraga H (1974) Intermolecular potentials from crystal data. III. Determination of empirical potentials and application to the packing configurations and lattice energies in crystals of hydrocarbons, carboxylic acids, amines, and amides. J Phys Chem 78(16):1595–1620. 10.1021/j100609a005

[24] Cole JC, Korb O, McCabe P, Read MG, Taylor R (2018) Knowledge-based conformer generation using the Cambridge structural database. J Chem Inf Model 58(3):615–629. 10.1021/acs.jcim.7b00697, pMID: 29425456 10.1021/acs.jcim.7b00697 29425456

[25] Liu DC, Nocedal J (1989) On the limited memory BFGS method for large scale optimization. Math Program 45(1):503–528. 10.1007/BF01589116

[26] Bell B (2015) CppAD. A C++ algorithmic differentiation package

[27] Allen FH, Bruno IJ (2010) Bond lengths in organic and metal-organic compounds revisited: X-H bond lengths from neutron diffraction data. Acta Crystallogr B 66(3):380–386. 10.1107/S0108768110012048 20484809 10.1107/S0108768110012048

[28] Otero-De-La-Roza A, Johnson ER (2012) A benchmark for non-covalent interactions in solids. J Chem Phys 137(5). 10.1063/1.4738961 10.1063/1.4738961 22894328

[29] Gavezzotti A, Presti LL, Rizzato S (2022) Molecular dynamics simulation of organic materials: structure, potentials and the MiCMoS computer platform. CrystEngComm 24:922–930. 10.1039/D1CE01360B

[30] Chisholm JA, Motherwell S (2005) COMPACK: a program for identifying crystal structure similarity using distances. J Appl Crystallogr 38(1):228–231. 10.1107/S0021889804027074

[31] Sacchi P, Lusi M, Cruz-Cabeza AJ, Nauha E, Bernstein J (2020) Same or different - that is the question: identification of crystal forms from crystal structure data. CrystEngComm 22:7170–7185. 10.1039/D0CE00724B

[32] Mayo RA, Otero-de-la Roza A, Johnson ER (2022) Development and assessment of an improved powder-diffraction-based method for molecular crystal structure similarity. CrystEngComm 24:8326–8338. 10.1039/D2CE01080A

[33] Beran GJO, Wright SE, Greenwell C, Cruz-Cabeza AJ (2022) The interplay of intra- and intermolecular errors in modeling conformational polymorphs. J Chem Phys 156(10):104112. 10.1063/5.0088027 35291791 10.1063/5.0088027

[34] Sacchi P, Wright SE, Neoptolemou P, Lampronti GI, Rajagopalan AK, Kras W, Evans CL, Hodgkinson P, Cruz-Cabeza AJ (2024) Crystal size, shape, and conformational changes drive both the disappearance and reappearance of ritonavir polymorphs in the mill. Proc Natl Acad Sci 121(15):e2319127121. 10.1073/pnas.2319127121 38557191 10.1073/pnas.2319127121 PMC11009673

[35] Beran GJ, Sugden IJ, Greenwell C, Bowskill DH, Pantelides CC, Adjiman CS (2022) How many more polymorphs of ROY remain undiscovered. Chem Sci 13(5):1288–1297. 10.1039/D1SC06074K 35222912 10.1039/d1sc06074k PMC8809489

[36] Sacchi P, Reutzel-Edens SM, Cruz-Cabeza AJ (2021) The unexpected discovery of the ninth polymorph of tolfenamic acid. CrystEngComm 23:3636–3647. 10.1039/D1CE00343G

[37] Sadiq G, Sharma S, Stevens JS, Martinez-Bulit P, Hunnisett LM, Cameron C, Samas B, Hawking E, Francia N, Lengyel J, Pidcock E, Rahman S, Nisbet M, Back K, Doherty C, Basford P, Cooper TG, O’Connor G, Bhardwaj RM (2025) An integrated approach combining experimental, informatics and energetic methods for solid form derisking of PF-06282999. J Pharm Sci 114(1):371–382. 10.1016/j.xphs.2024.10.013 39424195 10.1016/j.xphs.2024.10.013

[38] Chattopadhyay A, Hill AR, Wright SE, Beran GJ, Cruz-Cabeza AJ (2025) Lattice energy partitions in crystals of flexible molecules and the 40% limit. J Am Chem Soc. 10.1021/jacs.5c09997 41094351 10.1021/jacs.5c09997 PMC12576777

