# Supplementary material for: A benchmark study of force fields implemented in CSD software
Source: Struct Chem. 2026 Jan 10;37(3):1063–77. doi: 10.1007/s11224-025-02715-z (PMC13179903; doi:10.1007/s11224-025-02715-z)
Supplement: Supplementary file 1 — (1.30 MB) [file 11224_2025_2715_MOESM1_ESM.pdf]

Supplementary Information: A benchmark study of force fields implemented in CSD software

Lily M. Hunnisett[1*], Pietro Sacchi[1*] and Andrew G. P. Maloney[1*]

[1]The Cambridge Crystallographic Data Centre, 12 Union Road, Cambridge, CB2 1EZ, UK.

*Corresponding author(s). E-mail(s): lhunnisett@ccdc.cam.ac.uk; psacchi@ccdc.cam.ac.uk; maloney@ccdc.cam.ac.uk;

Contents

1 CG2019 Dataset preparation **3**

2 Force fields: functional forms **3**
2.1 CLP . . . 3
2.2 UNI and CSD-OPCS16 . . . 4
2.3 DreidingII and Momany . . . 4

3 Comparison of Crystal Optimiser scores and VisualHabit lattice energies **4**

4 Crystal structure optimisation **6**
4.1 Comparison methods . . . 6
4.1.1 Crystal structure . . . 6
4.1.2 Molecular geometries . . . 6
4.2 Comparison results . . . 7
4.2.1 Crystal structures: CLP (constrained optimisation) . . . 8
4.2.2 Crystal structures: CLP (full optimisation) . . . 9
4.2.3 Crystal structures: UNI (constrained optimisation) . . . 13
4.2.4 Crystal structures: UNI (full optimisation) . . . 15
4.2.5 Crystal structures: CSD-OPCS16 (constrained optimisation) . . 19
4.2.6 Crystal structures: CSD-OPCS16 (full optimisation) . . . 20
4.2.7 Crystal structures: DreidingII (constrained optimisation) . . . 24
4.2.8 Crystal structures: DreidingII (full optimisation) . . . 26
4.2.9 Crystal structures: Momany (constrained optimisation) . . . 30
4.2.10 Crystal structures: Momany (full optimisation) . . . 31
4.2.11 Molecular geometries: CLP . . . 35
4.2.12 Molecular geometries: UNI . . . 37
4.2.13 Molecular geometries: CSD-OPCS16 . . . 38
4.2.14 Molecular geometries: DreidingII . . . 40
4.2.15 Molecular geometries: Momany . . . 41

5 Comparison of lattice energies with experimental sublimation enthalpies **42**
5.1 Original CLP vs CSD-CLP . . . 42
5.2 Lattice energy results by compound class . . . 43

6 Polymorphic dataset **47**
6.1 Ritonavir 47
6.2 ROY 48
6.3 Tolfenamic acid (TFA) 49
6.4 PF-06282999 50

1 CG2019 Dataset preparation

The crystal structures used for our calculations were selected according to the CSD refcodes reported in the original publication of Chickos and Gavezzotti [1] except for six cases which are reported in Table 2. Two CSD entries had an updated refcode, one was not found in the database. The remaining structures were those of the deuterated compound. In these cases, have decided to use the non-deuterated structures instead. When a change of structure was needed, we have made sure to use the same criteria as Chickos and Gavezzotti when selecting alternatives, namely, selecting structures with the lowest R-factor and/or with diffraction data collected close to room temperature.

Supplementary Table 1 CSD refcode changes from CG2019 paper.

Previous refcode	New refcode	Reason
NAPHTA10	NAPHTA52	changed
OCTANE01	OCTANE12	changed
HEPTAN02	HEPTAN03	not found
DUCKOB03	DUCKOB04	deuterated
THIOUR16	THIOUR20	deuterated
ZZZITY02	ZZZITY01	deuterated

Supplementary Table 2 List of structures for which calculations failed and associated reason.

CSD refcode	FF affected	Module affected	Reason
TROXAN	all	*CrystalOptimiser*	space group setting not recognised
TCYMET	all	*CrystalOptimiser*	space group setting not recognised
BISDOY	all	*VisualHabit*	problem related with disorder in CSD entry
ZZZVCO04	all	*VisualHabit*	problem related with disorder in CSD entry
ZZZIVG02	all	*VisualHabit*	problem related with disorder in CSD entry
CUVJUY	CLP	both	atom typing problem

2 Force fields: functional forms

The general form of the functionals used to model interatomic potentials between atoms i and j, each in different non-bonded molecules, are reported in this section.

2.1 CLP

The Coulomb-London-Pauli (CLP) interatomic potential, which is mainly described in [2], is calculated as:

$$U_{ij} = \frac{q_i q_j}{4\pi\varepsilon_0} r_{ij}^{-1} - F_P P_{ij} r^{-4} - F_D D_{ij} r_{ij}^{-6} + F_R T_{i,j} r_{ij}^{-12}$$

The first term is the coulombic term, where the atomic partial charge q_i is calculated from the atomic Extended Hückel charge [3], q_i^0, as $q_i = F_Q q_i^0$, where F_Q is a scaling parameter (typically 0.414).

The remaining terms are the polarisation term (r^{-4}), the dispersion term (r^{-6}) and the repulsion term (r^{-12}). P_{ij} is a parameter that depends on atom polarisability, as well as atomic charges; D_{ij} depends on both polarisability and ionisation potentials; T_{ij} is related to atomic diffusion and the number of valence electrons. F_P, F_D and F_R are scaling parameters. Additional information about CLP parameters can also be found in the user manual for the MiCMoS platform [4].

2.2 UNI and CSD-OPCS16

Both the UNI[5–7] and CSD-OPCS16[8] force fields calculate interatomic interactions using a 6-exp potential of the form:

$$U_{ij} = A\exp(-Br_{ij}) - Cr_{ij}^{-6}$$

The main difference between these two force fields is the values for the parameters A, B and C. For CSD-OPCS16, these parameters are based on the parameters for UNI, but were tuned with the aim of reproducing CSD structures for application to crystal structure prediction methods (see [8]).

2.3 DreidingII and Momany

DreidingII[9] and Momany[10] have the same general functional form, comprising a coulombic term (r^{-1}) and attractive (r^{-6}) and repulsive (r^{-12}) van der Waals terms.

$$U_{ij} = \frac{q_i q_j}{4\pi\varepsilon_0} r_{ij}^{-1} - A_{ij} r_{ij}^{-6} + B_{ij} r_{ij}^{-12}$$

At variance with Momany, DreidingII also includes an additional term for groups of atoms that are involved in hydrogen bonds. This term is formed by an attractive term (r^{-10}) and a repulsive one (r^{-12}), and it depends on the distance between donor and acceptor atoms (r_{DA}), as well as the hydrogen bond angle θ:

$$U_{hb} = (D_1 r_{DA}^{-12} - D_2 r_{DA}^{-10}) \cos^4\theta$$

Thus, the total energy for the interaction of two different in molecules in DreidingII corresponds to the sum of interatomic potentials and this hydrogen bond term:

$$E_{mol-mol} = \sum U_{ij} + \sum U_{hb}$$

For this work, Gasteiger charges [11] were used for Momany and DreidingII, although different charge models can also be used with these force fields.

3 Comparison of Crystal Optimiser scores and VisualHabit lattice energies

As mentioned in the main text, *Crystal Optimiser* calculations try to minimise a score, Z_{total}, by optimising the position of molecules in the crystal and/or by optimising their geometry using a knowledge-based method (KBF) [8].

This score is composed of two terms:

$$Z_{total} = Z_{inter}^{FF} + \kappa Z_{intra}^{KBF}$$

If the molecule geometry is kept fixed during optimisation, the final optimiser score will only be composed of the intermolecular term Z_{inter}^{FF}, which accounts for the stabilisation of the crystal structure stemming from intermolecular interactions only. If the molecule geometry is changed, however, the additional term Z_{intra}^{KBF}, scaled by a factor κ, is also added. Since the score Z_{intra}^{KBF} is calculated by comparing molecular geometries with statistical values from the CSD database, its value will not directly represent an energy. Because of this, Z_{total} scores cannot be compared directly to sublimation enthalpies, i.e., they should not be considered as a direct indication of the stability of a crystal structure (in strict thermodynamic terms). Even so, it is not unreasonable to expect that these scores should be proportional to the lattice energies calculated using the same force fields but with different methods, like *VisualHabit*, for example, as we have done here.

Table 3 shows the result for the comparison of *Crystal Optimiser* Z_{total} scores with lattice energies calculated using *VisualHabit* for the structures of the CG2019 dataset. Our results show that, if molecule geometries are not optimised, the optimiser score closely resembles the lattice energy. The results for the CSD-OPCS16 force field show a systematic deviation, whose possible causes are under investigation. If molecular

geometries are also optimised, all force fields show a similar deviation from the lattice energies, which is the result of the addition of the scaled intramolecular term.

We believe that these results justify our decision to only use *VisualHabit* lattice energies for the comparison with experimental sublimation enthalpies presented in this work.

Supplementary Table 3 Mean Absolute Error (MAE), RMSD, average error (AVG. ERR.), and maximum absolute error (MAX) for the comparison of *CrystalOptimiser* scores with *VisualHabit* lattice energies after structure optimisation. Units can be loosely interpreted as energies in kJ mol^{-1} (see discussion in the text), except for percentage errors.

**Calculation type**	**Forcefield**	**MAE**	**RMSD**	**AVG. ERR. (%)**	**MAX**
Constrained opt	CLP	0.11	0.48	0.13	6.05
	UNI	0.08	0.09	0.08	0.44
	CSD-OPCS16	3.43	3.73	3.69	10.26
	DreidingII	0.11	0.48	0.13	6.05
	Momany	0.18	1.48	0.21	32.51
Full opt	CLP	5.83	10.89	4.72	68.15
	UNI	5.73	10.77	4.81	67.50
	CSD-OPCS16	5.96	9.72	6.04	62.73
	DreidingII	5.65	10.39	5.47	57.61
	Momany	6.36	11.63	6.56	68.31

4 Crystal structure optimisation

This section contains additional details about the results of *CrystalOptimiser* for the CG2019 dataset. For each entry, the CSD crystal structure was used as reference, and various descriptors were calculated.

4.1 Comparison methods

4.1.1 Crystal structure

The overall similarity between the optimised and reference structures was assessed using the COMPACK method [12] as implemented in the CSD Python API, using a cluster of 20 molecules and default tolerances (20% for distances and 20° for angles). This method returns the number of molecules that overlap between the compared structures (N_{match}), as well as the root mean squared deviation (RMSD-20, in Å) for the comparison. Thus, a larger value of N_{match} and a small RMSD-20 indicate a high similarity between the compared structures [13].

For full geometry optimisation calculations, the difference between the reference and optimised unit cells was also assessed by calculating:

- relative differences for the cell axes lengths (shown below for the a axis)

$$a_{rel} = \frac{|a_{OPT} - a_{CSD}|}{a_{CSD}}$$

- root mean squared deviation for the cell axes lengths (RMSD-CL)

$$RMSD - CL = \frac{\sqrt{(a_1 - a_2)^2 + (b_1 - b_2)^2 + (c_1 - c_2)^2}}{3}$$

- root mean squared deviation for the cell axes angles (RMSD-CA)

$$RMSD - CA = \frac{\sqrt{(\alpha_1 - \alpha_2)^2 + (\beta_1 - \beta_2)^2 + (\gamma_1 - \gamma_2)^2}}{3}$$

We note that a new space group and/or unit cell may be assigned by *CrystalOptimiser* after each calculation, if the original structure has been distorted enough. In such cases, the above descriptors cease to be useful, and only RMSD-20 and N_{match} can be used for the comparison.

Supplementary Table 4 Average unit cell distortion descriptors for N fully optimised structures, as compared to the corresponding CSD entries.

**Force field**	**RMSD-CL (Å)**	**RMSD-CA (°)**	**rel. change** a axis	**rel. change** b axis	**rel. change** c axis
CLP	0.34	1.89	0.048	0.027	0.034
UNI	0.30	1.58	0.042	0.021	0.030
CSD-OPCS16	0.26	1.45	0.037	0.016	0.027
Dreiding II	0.32	1.69	0.044	0.022	0.032
Momany	0.26	1.62	0.039	0.018	0.024

4.1.2 Molecular geometries

Molecular geometry for fully-optimised crystal structures in the CG2019 dataset were compared to corresponding orginal CSD entries by analysing the differences in atomic positions (RMSD-POS, in Å), in bond angles (RMSD-BA, in °) and torsion angles (RMSD-TOR, in °):

Supplementary Table 5 Number of structures in CG2019 dataset for which space group changed after optimisation. N is the total number of structures calculated for each force field.

Force field	N	Constrained opt	Full Opt
**CLP**	663	181	181
**UNI**	664	182	182
**CSD-OPCS16**	664	182	182
**DreidingII**	664	182	182
**Momany**	550	155	155

Supplementary Table 6 Number of structures showing large volume variation as a result of unit cell reassignment after optimisation and corresponding average COMPACK N_match. N is the total number of structures calculated for each force field.

force field	N	Constrained opt	N_match	Full Opt	N_match
**CLP**	663	5	20	5	19.8
**UNI**	664	5	20	6	18.7
**CSD-OPCS16**	664	5	20	7	18.4
**DreidingII**	664	5	20	5	20
**Momany**	550	2	20	3	16.3

- RMSD-POS values were calculated using the Molceule Overlay feature available to CSD-Materials users.
- RMSD-BA and RMSD-TOR were calculated as:

$$RMSD - BA \ or \ RMSD - TOR = \frac{\sqrt{\sum (x_{OPT} - x_{CSD})^2}}{N_{obs}}$$

4.2 Comparison results

This section reports plots showing descriptors for the comparison of optimised crystal structures and molecular geometries for the CG2019 dataset with the corresponding CSD entries. Results are reported for each of the considered force fields (CLP, UNI, CSD-OPCS16, DreidingII and Momany). Comparison of molecular geometries was performed only for the case of fully optimised structures, since for constrained optimisations the molecular geometry is kept fixed.

4.2.1 Crystal structures: CLP (constrained optimisation)

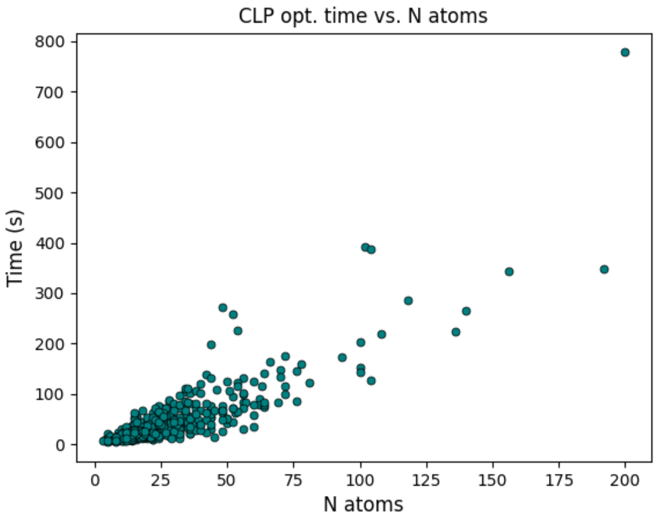

Supplementary Figure 1 Optimisation time vs. number of atoms for constrained optimisations using the CLP force field

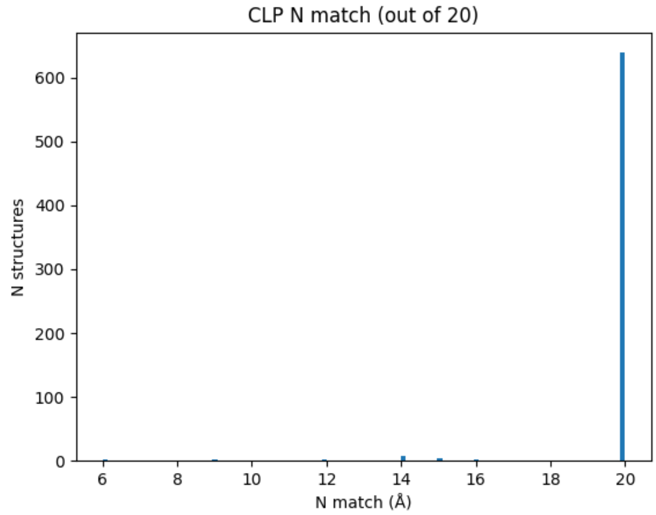

Supplementary Figure 2 Number of matched molecules for COMPACK clusters of 20 molecules for constrained optimisations using the CLP force field

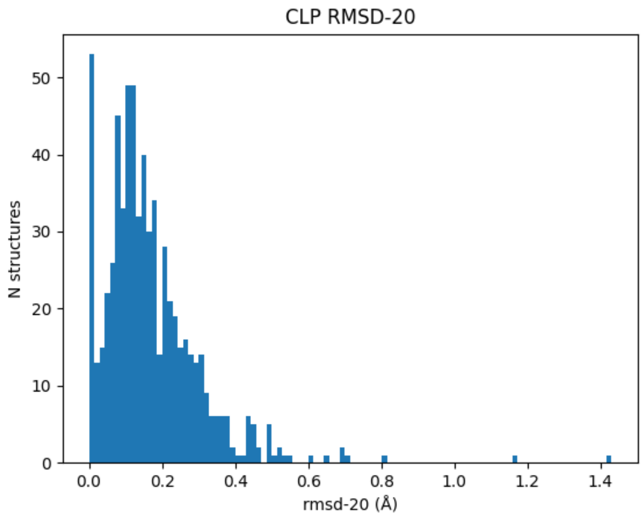

Supplementary Figure 3 RMSD-20 values (in Å) for COMPACK clusters of 20 molecules for constrained optimisations using the CLP force field

4.2.2 Crystal structures: CLP (full optimisation)

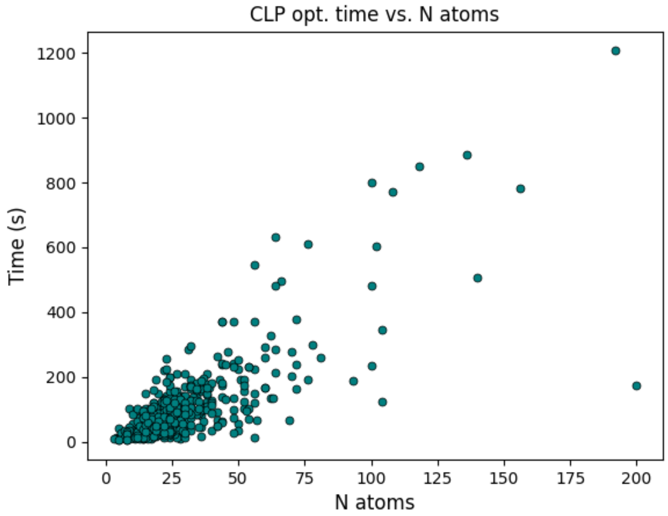

Supplementary Figure 4 Optimisation time vs. number of atoms for full optimisations using the CLP force field

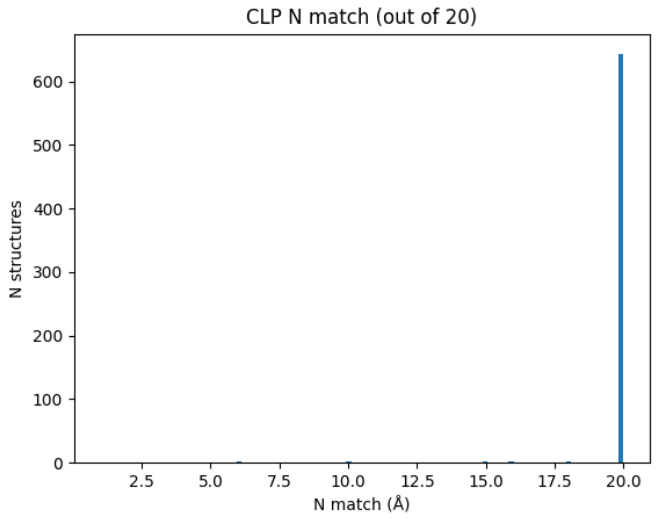

Supplementary Figure 5 Distribution of the number of matched molecules for COMPACK clusters of 20 molecules for full optimisations using the CLP force field

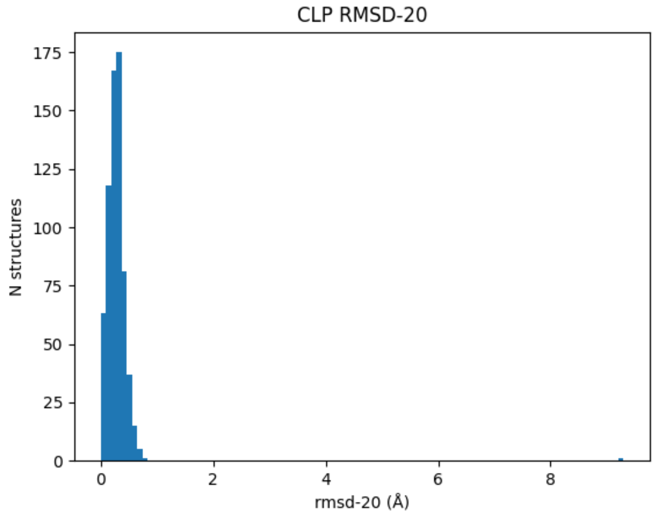

Supplementary Figure 6 Distribution of RMSD-20 values (in Å) for COMPACK clusters of 20 molecules for full optimisations using the CLP force field

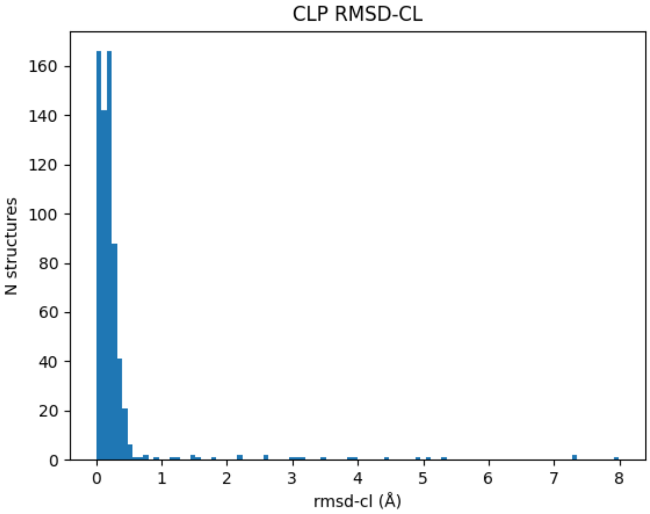

Supplementary Figure 7 Distribution of RMSD values for unit cell axes lengths (RMSD-CL, in Å) for full optimisations using the CLP force field

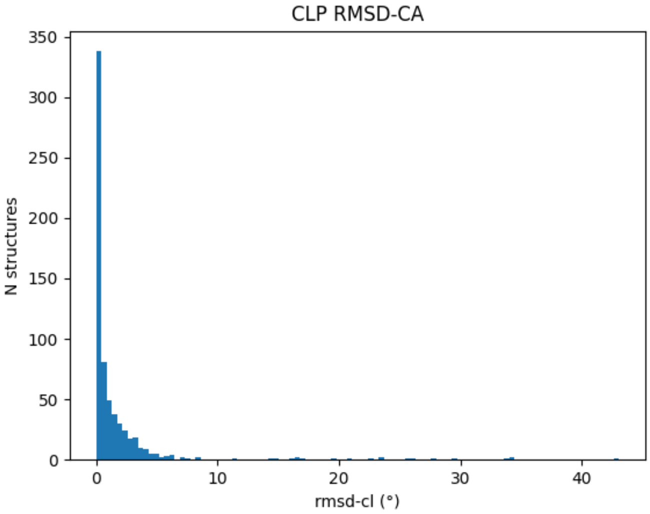

Supplementary Figure 8 Distribution of RMSD values for unit cell angles (RMSD-CA, in °) for full optimisations using the CLP force field

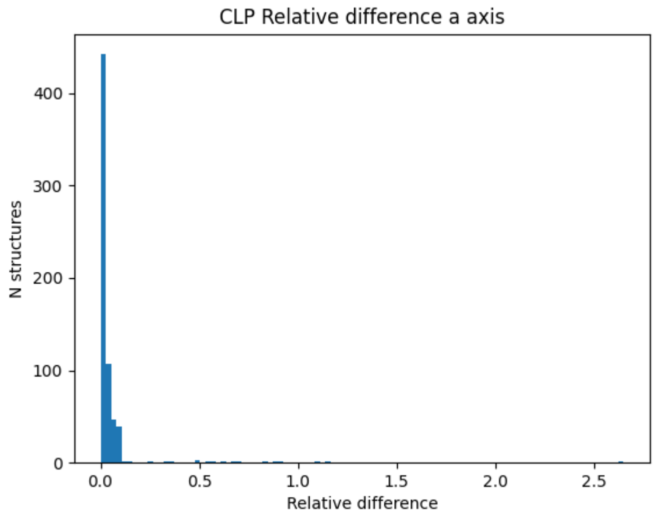

Supplementary Figure 9 Distribution of relative a axis values (with reference to corresponding CSD entry) for full optimisations using the CLP force field

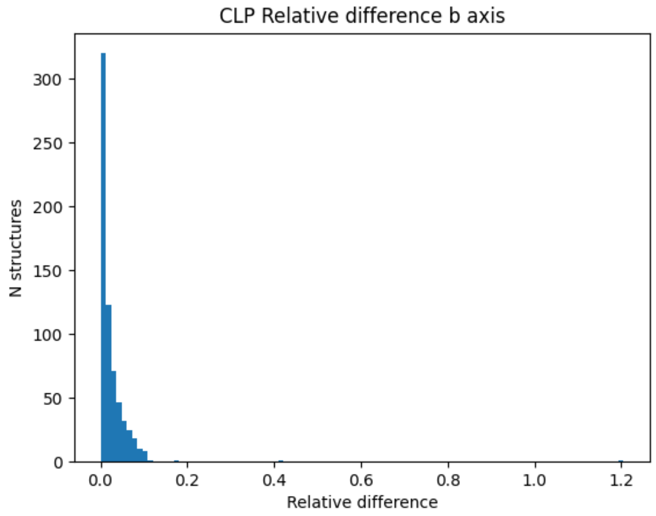

Supplementary Figure 10 Distribution of relative b axis values (with reference to corresponding CSD entry) for full optimisations using the CLP force field

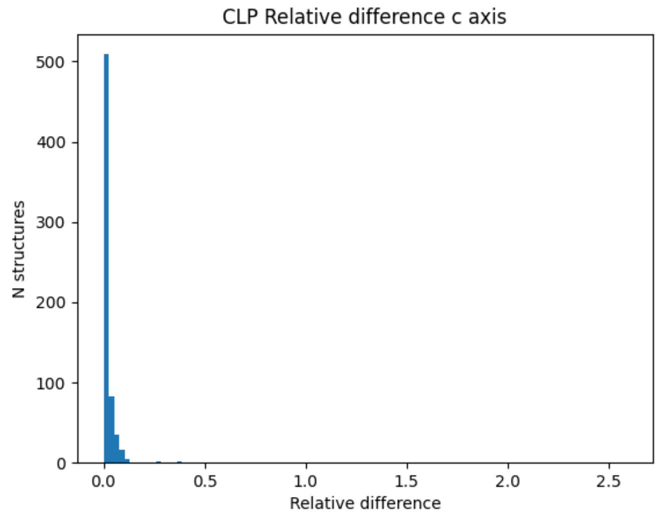

Supplementary Figure 11 Distribution of relative c axis values (with reference to corresponding CSD entry) for full optimisations using the CLP force field

4.2.3 Crystal structures: UNI (constrained optimisation)

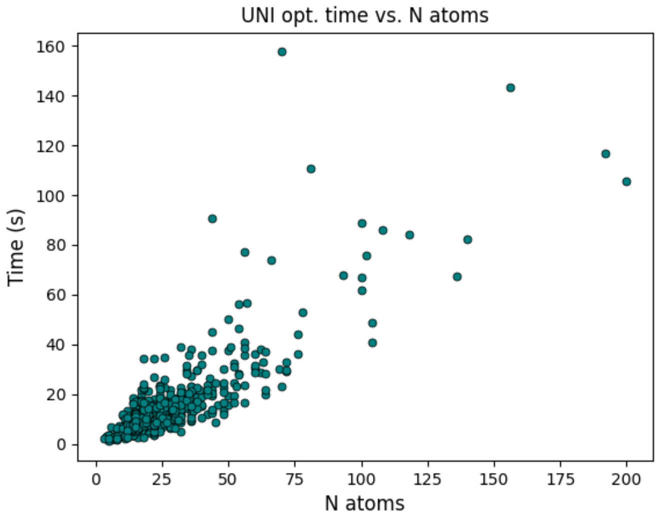

Supplementary Figure 12 Optimisation time vs. number of atoms for constrained optimisations using the UNI force field

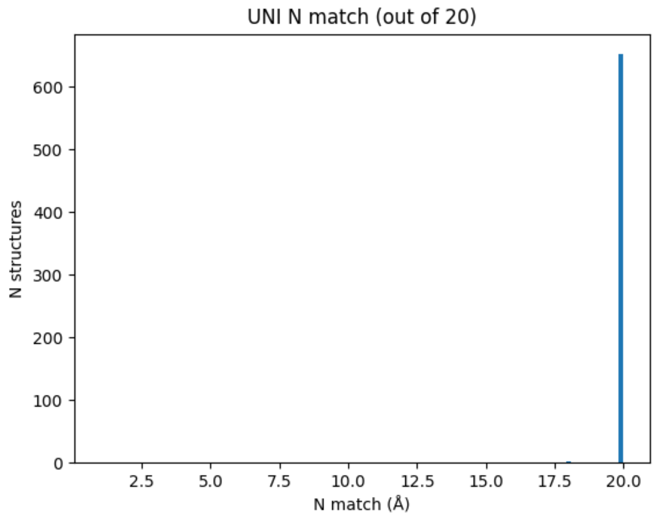

Supplementary Figure 13 Number of matched molecules for COMPACK clusters of 20 molecules for constrained optimisations using the UNI force field

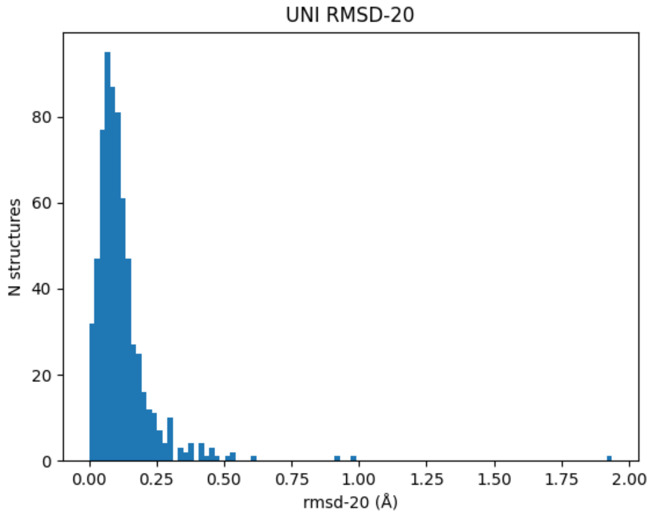

Supplementary Figure 14 RMSD-20 values (in Å) for COMPACK clusters of 20 molecules for constrained optimisations using the UNI force field

4.2.4 Crystal structures: UNI (full optimisation)

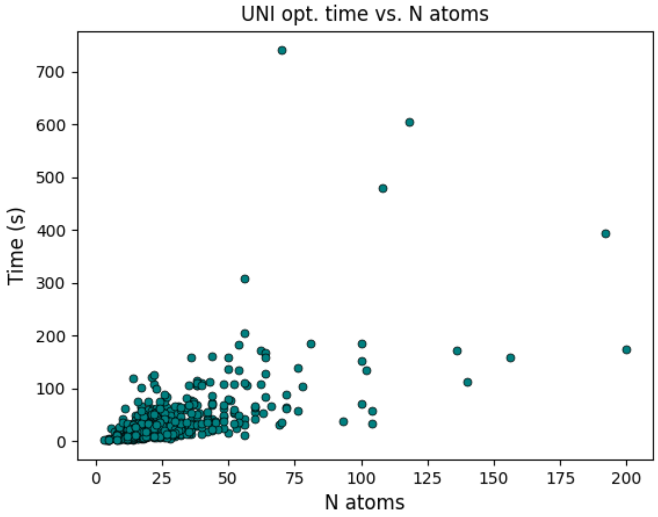

Supplementary Figure 15 Optimisation time vs. number of atoms for full optimisations using the UNI force field

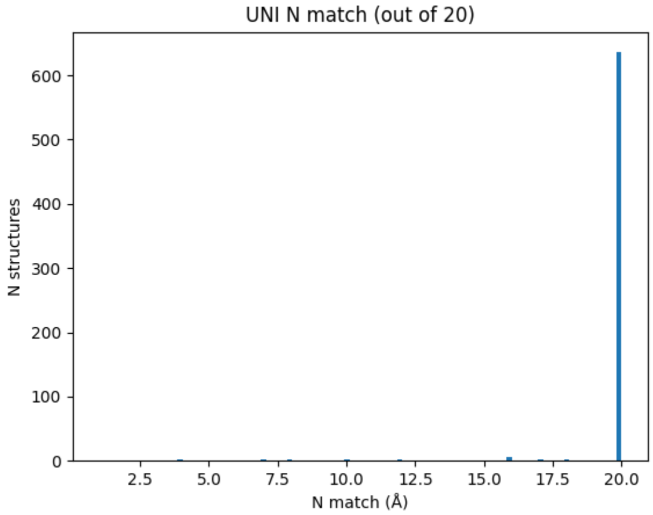

Supplementary Figure 16 Distribution of the number of matched molecules for COMPACK clusters of 20 molecules for full optimisations using the UNI force field

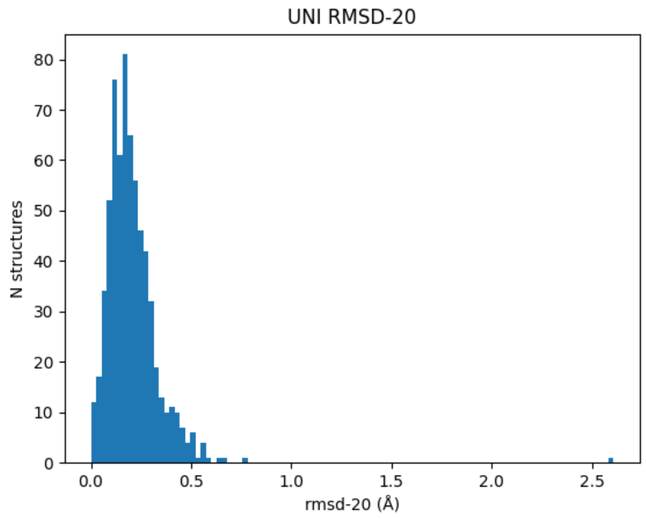

Supplementary Figure 17 Distribution of RMSD-20 values (in Å) for COMPACK clusters of 20 molecules for full optimisations using the UNI force field

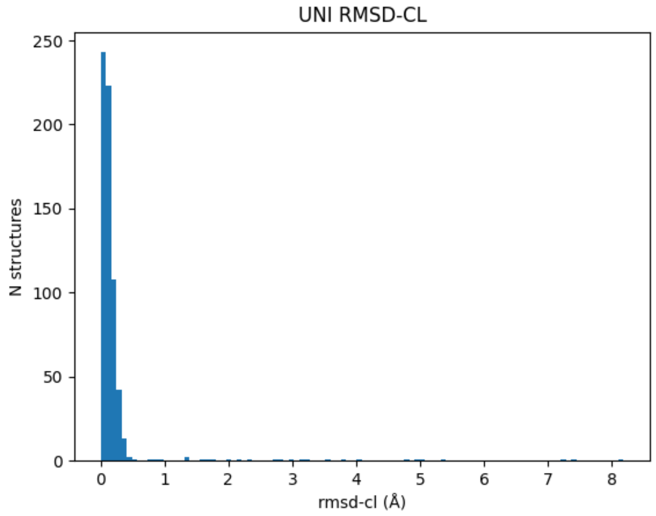

Supplementary Figure 18 Distribution of RMSD values for unit cell axes lengths (RMSD-CL, in Å) for full optimisations using the UNI force field

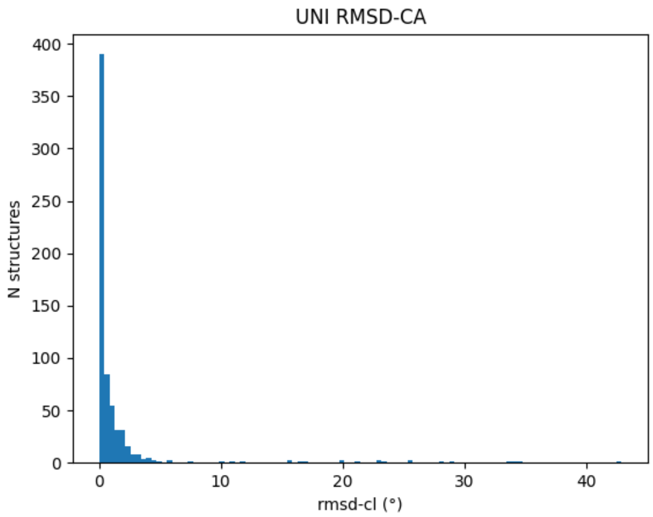

Supplementary Figure 19 Distribution of RMSD values for unit cell angles (RMSD-CA, in °) for full optimisations using the UNI force field

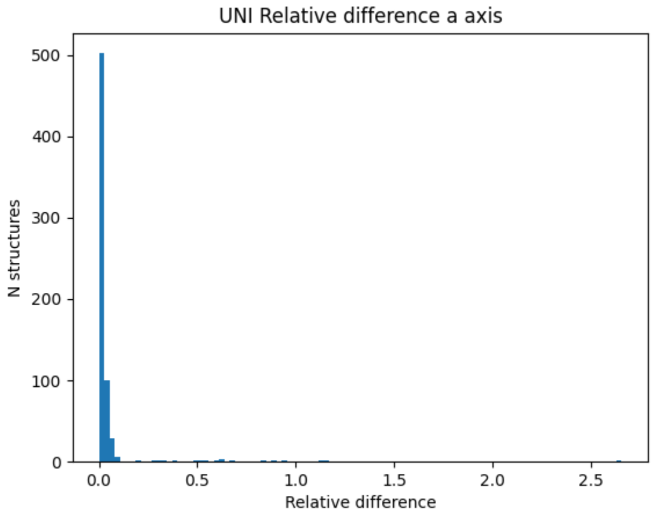

Supplementary Figure 20 Distribution of relative a axis values (with reference to corresponding CSD entry) for full optimisations using the UNI force field

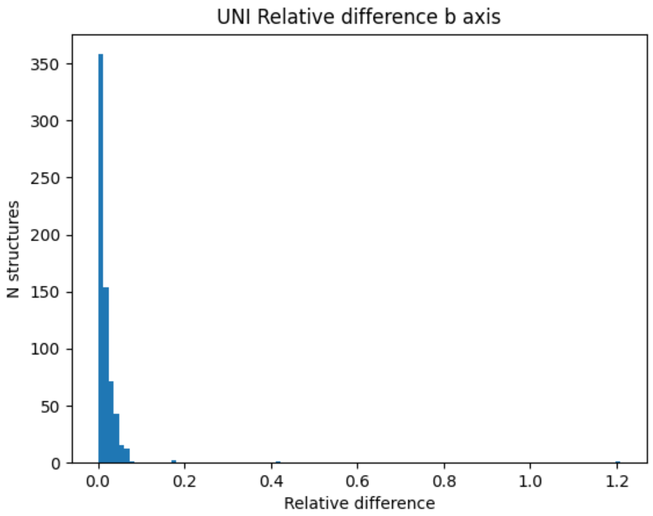

Supplementary Figure 21 Distribution of relative *b* axis values (with reference to corresponding CSD entry) for full optimisations using the UNI force field

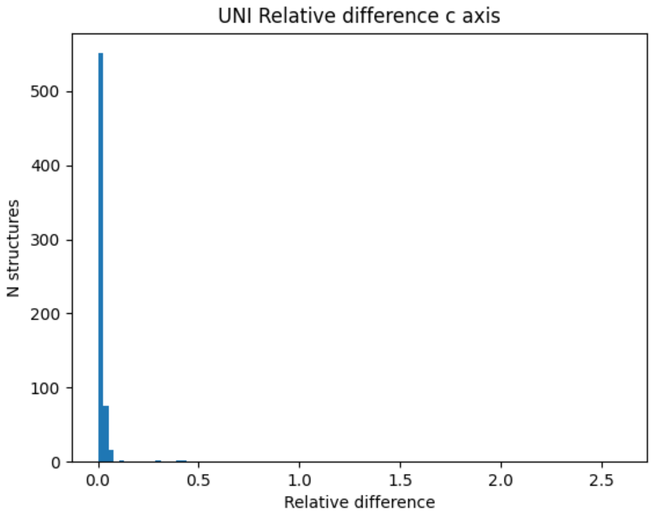

Supplementary Figure 22 Distribution of relative *c* axis values (with reference to corresponding CSD entry) for full optimisations using the UNI force field

4.2.5 Crystal structures: CSD-OPCS16 (constrained optimisation)

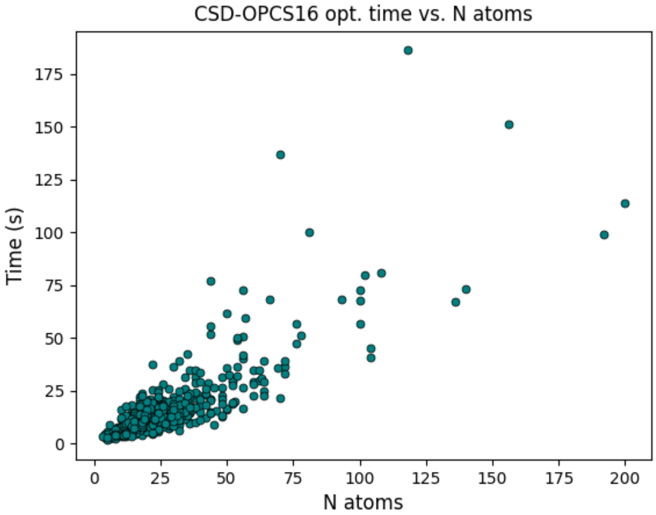

Supplementary Figure 23 Optimisation time vs. number of atoms for constrained optimisations using the CSD-OPCS16 force field

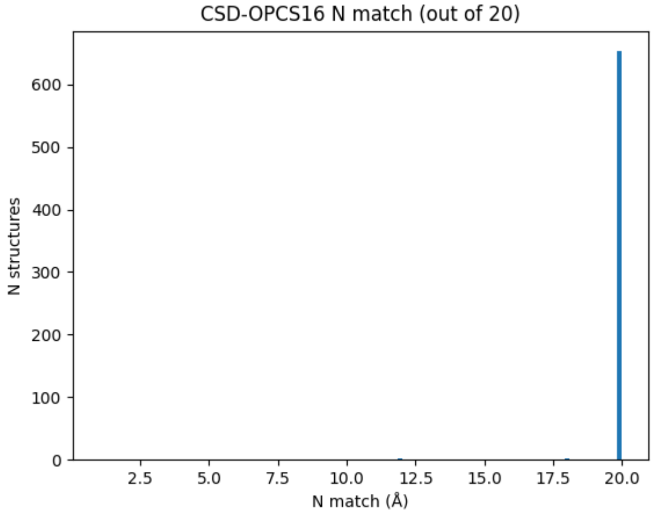

Supplementary Figure 24 Number of matched molecules for COMPACK clusters of 20 molecules for constrained optimisations using the CSD-OPCS16 force field

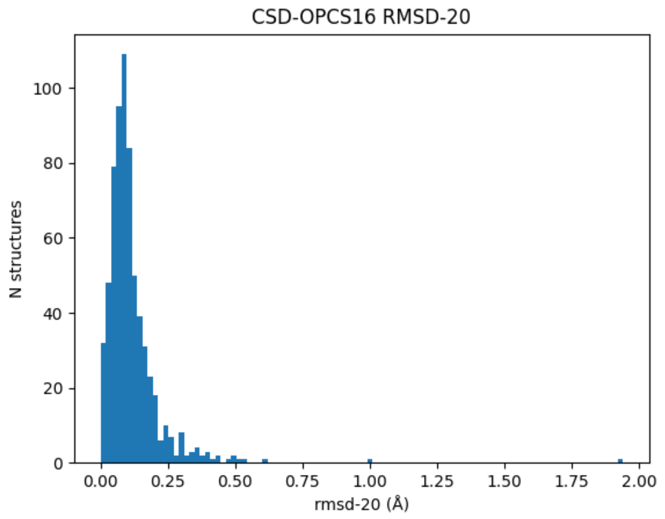

Supplementary Figure 25 RMSD-20 values (in Å) for COMPACK clusters of 20 molecules for constrained optimisations using the CSD-OPCS16 force field

4.2.6 Crystal structures: CSD-OPCS16 (full optimisation)

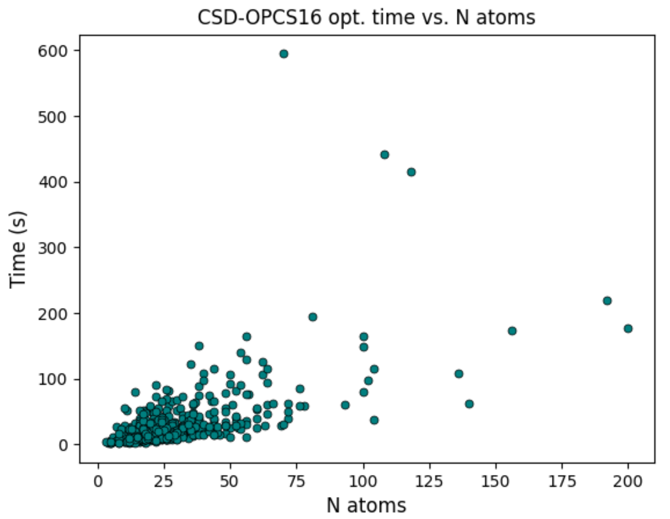

Supplementary Figure 26 Optimisation time vs. number of atoms for full optimisations using the CSD-OPCS16 force field

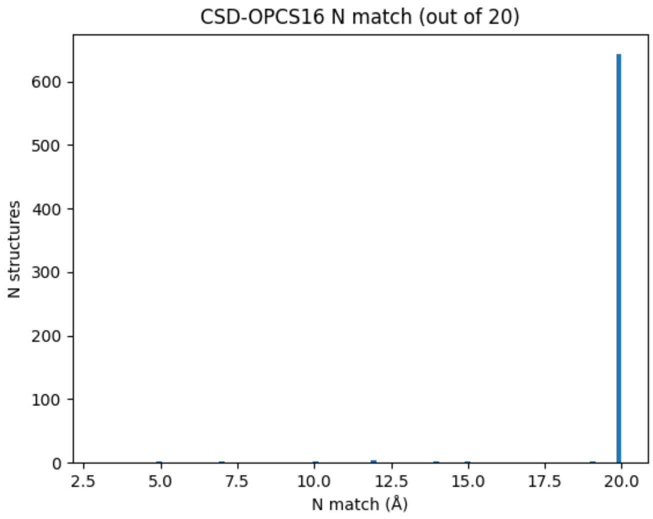

Supplementary Figure 27 Distribution of the number of matched molecules for COMPACK clusters of 20 molecules for full optimisations using the CSD-OPCS16 force field

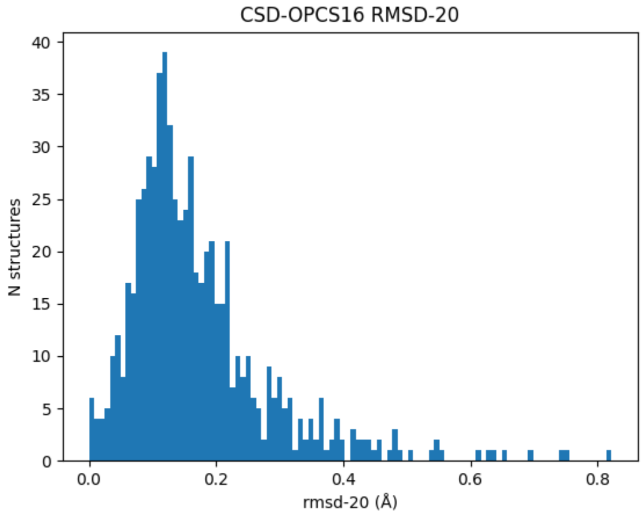

Supplementary Figure 28 Distribution of RMSD-20 values (in Å) for COMPACK clusters of 20 molecules for full optimisations using the CSD-OPCS16 force field

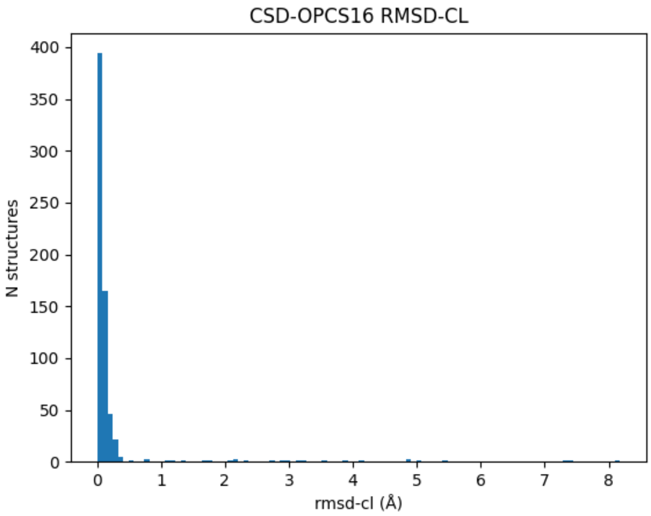

Supplementary Figure 29 Distribution of RMSD values for unit cell axes lengths (RMSD-CL, in Å) for full optimisations using the CSD-OPCS16 force field

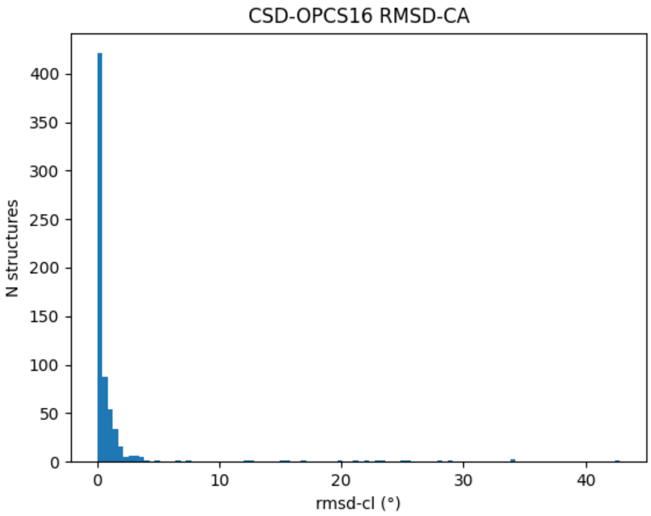

Supplementary Figure 30 Distribution of RMSD values for unit cell angles (RMSD-CA, in °) for full optimisations using the CSD-OPCS16 force field

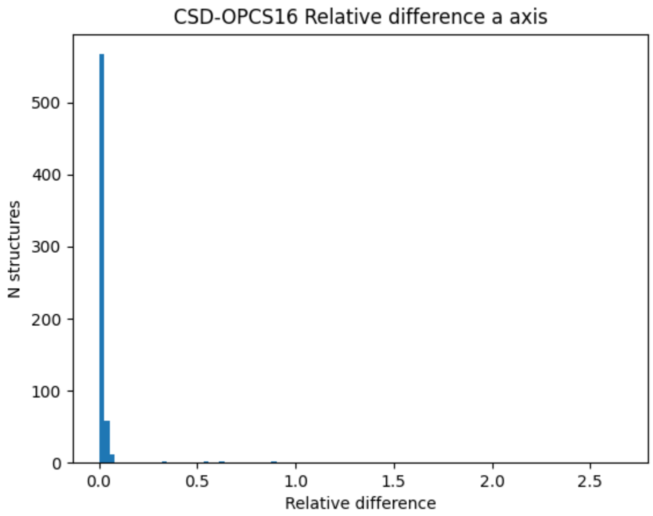

Supplementary Figure 31 Distribution of relative a axis values (with reference to corresponding CSD entry) for full optimisations using the CSD-OPCS16 force field

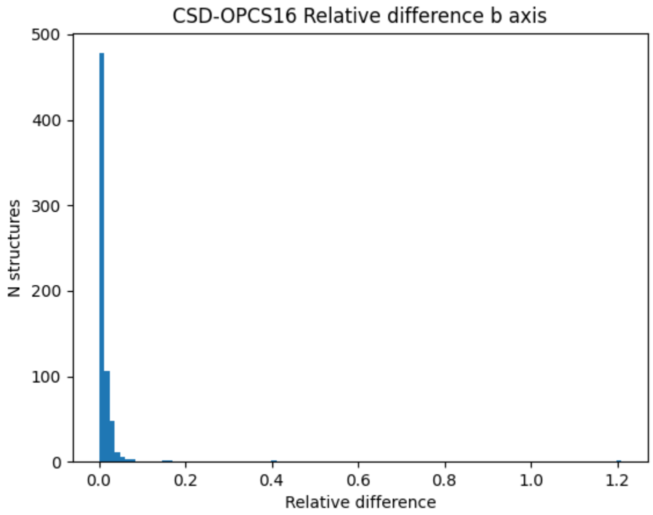

Supplementary Figure 32 Distribution of relative b axis values (with reference to corresponding CSD entry) for full optimisations using the CSD-OPCS16 force field

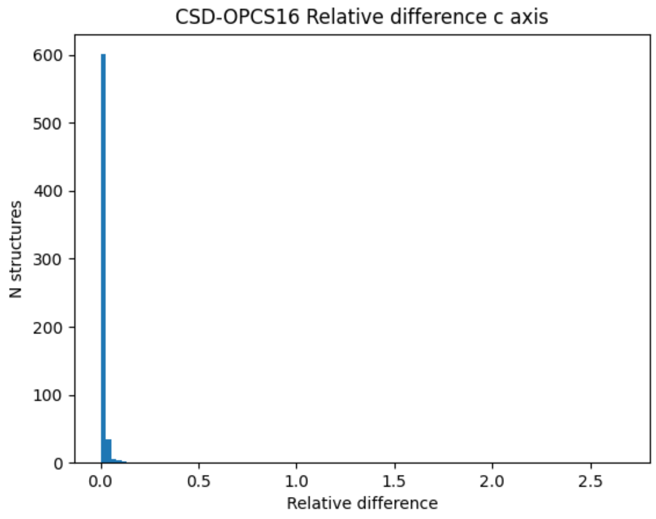

Supplementary Figure 33 Distribution of relative c axis values (with reference to corresponding CSD entry) for full optimisations using the CSD-OPCS16 force field

4.2.7 Crystal structures: DreidingII (constrained optimisation)

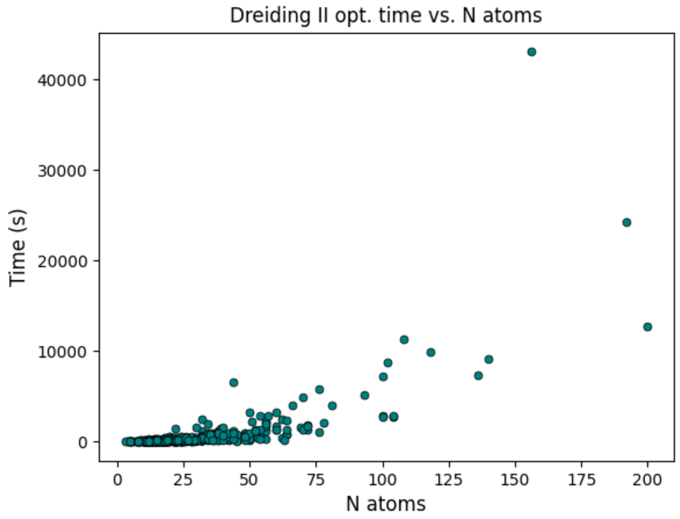

Supplementary Figure 34 Optimisation time vs. number of atoms for constrained optimisations using the DreidingII force field

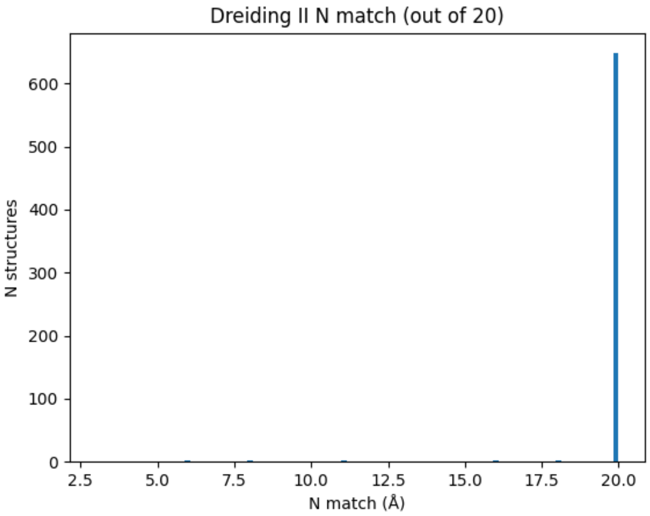

Supplementary Figure 35 Number of matched molecules for COMPACK clusters of 20 molecules for constrained optimisations using the DreidingII force field

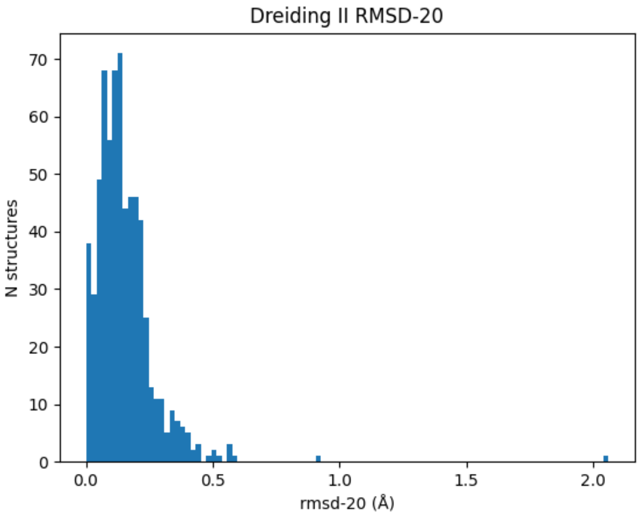

Supplementary Figure 36 RMSD-20 values (in Å) for COMPACK clusters of 20 molecules for constrained optimisations using the DreidingII force field

4.2.8 Crystal structures: DreidingII (full optimisation)

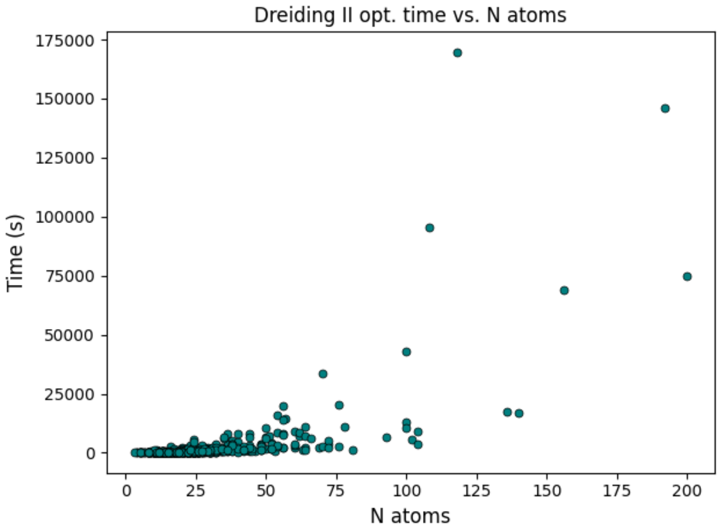

Supplementary Figure 37 Optimisation time vs. number of atoms for full optimisations using the DreidingII force field

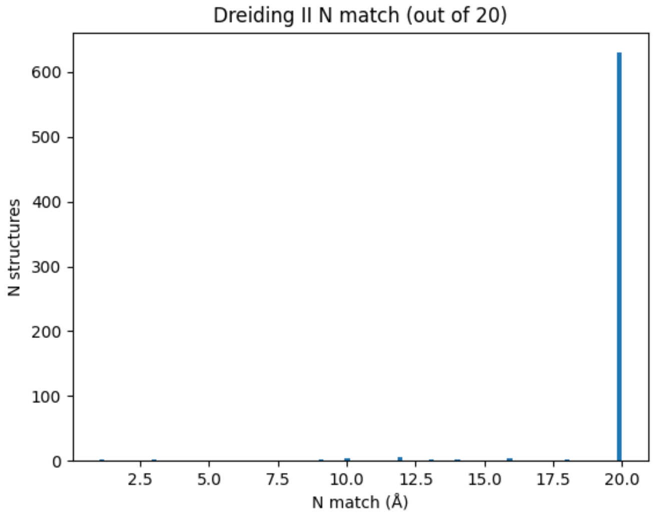

Supplementary Figure 38 Distribution of the number of matched molecules for COMPACK clusters of 20 molecules for full optimisations using the DreidingII force field

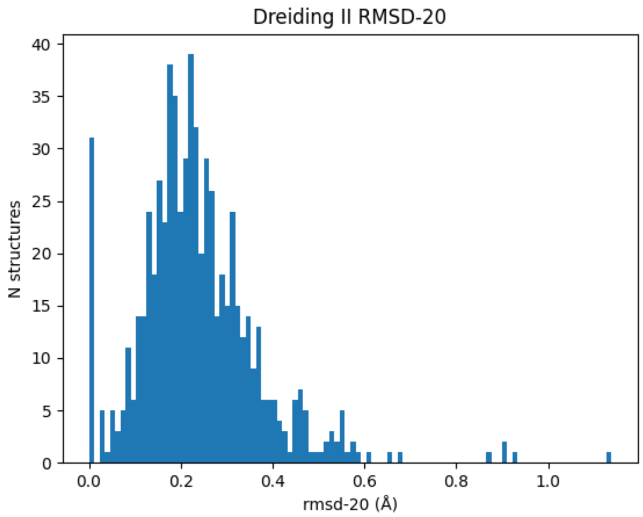

Supplementary Figure 39 Distribution of RMSD-20 values (in Å) for COMPACK clusters of 20 molecules for full optimisations using the DreidingII force field

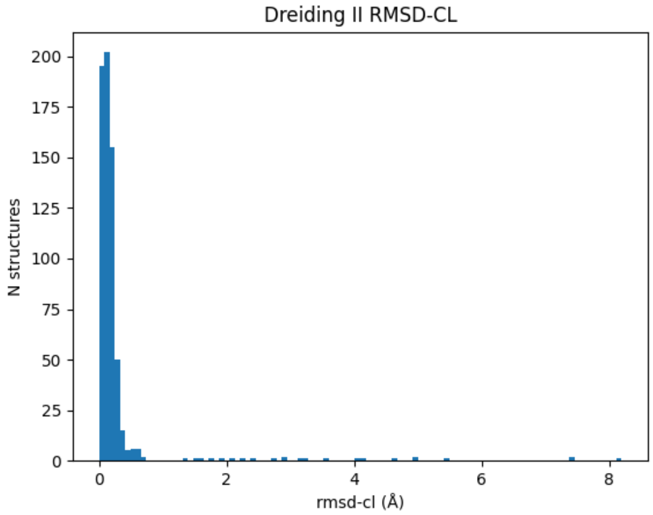

Supplementary Figure 40 Distribution of RMSD values for unit cell axes lengths (RMSD-CL, in Å) for full optimisations using the DreidingII force field

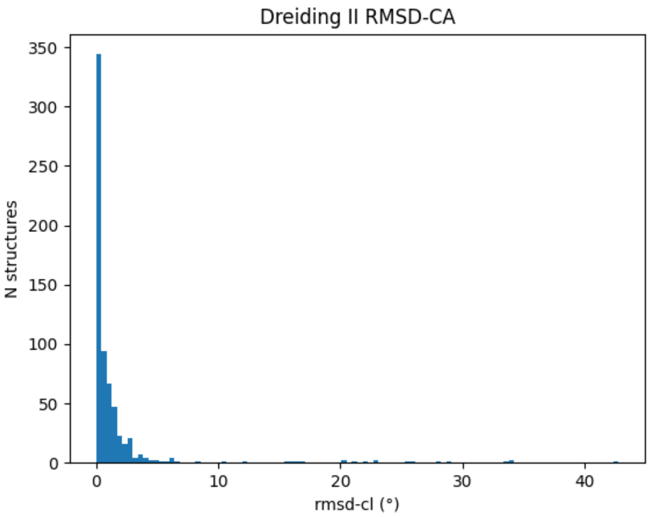

Supplementary Figure 41 Distribution of RMSD values for unit cell angles (RMSD-CA, in °) for full optimisations using the DreidingII force field

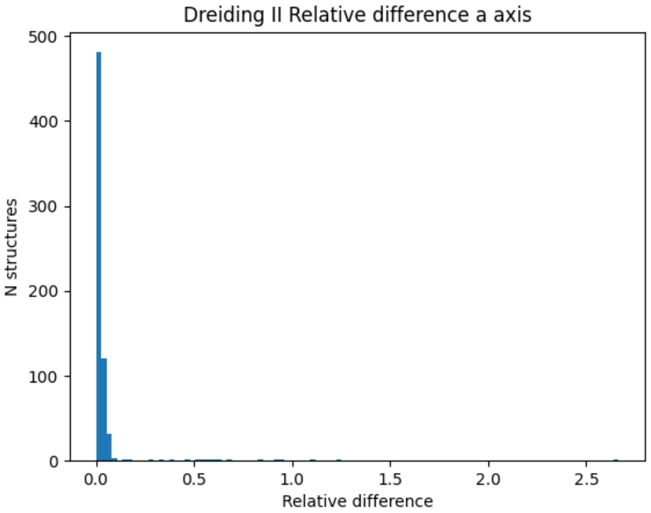

Supplementary Figure 42 Distribution of relative *a* axis values (with reference to corresponding CSD entry) for full optimisations using the DreidingII force field

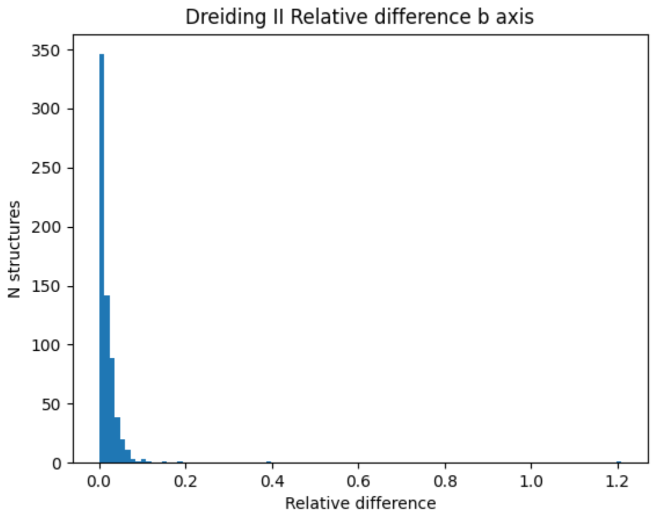

Supplementary Figure 43 Distribution of relative b axis values (with reference to corresponding CSD entry) for full optimisations using the DreidingII force field

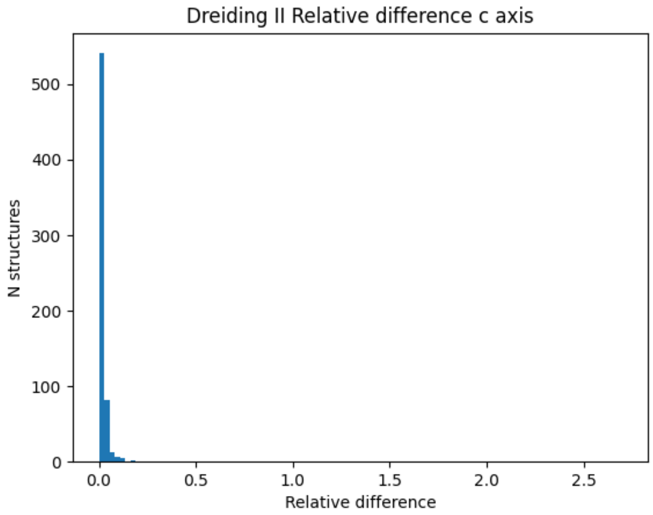

Supplementary Figure 44 Distribution of relative c axis values (with reference to corresponding CSD entry) for full optimisations using the DreidingII force field

4.2.9 Crystal structures: Momany (constrained optimisation)

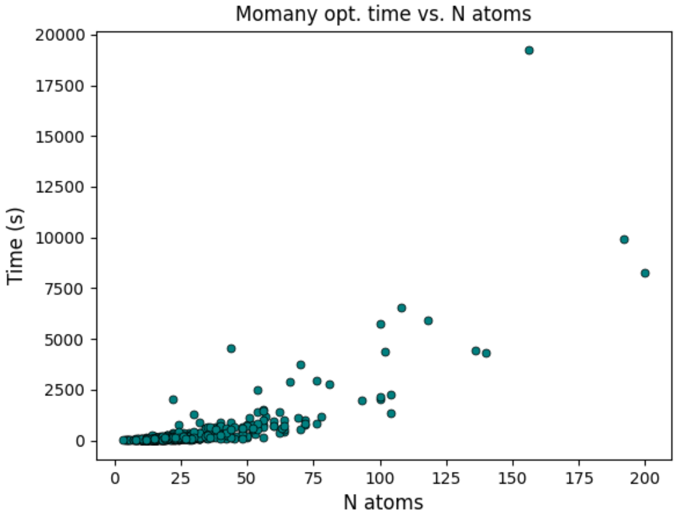

Supplementary Figure 45 Optimisation time vs. number of atoms for constrained optimisations using the Momany force field

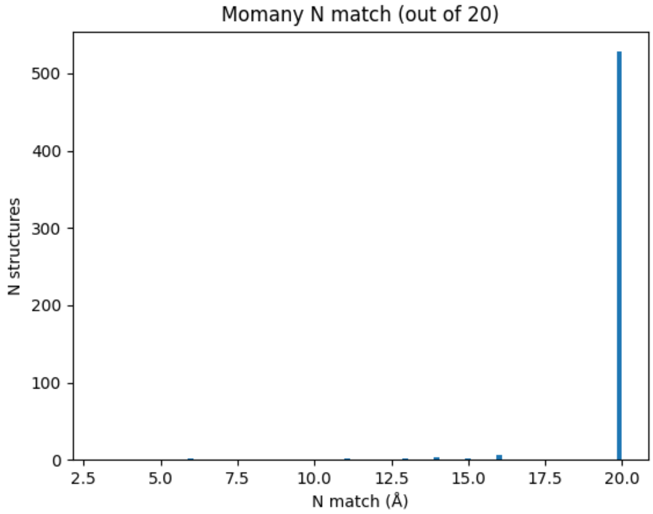

Supplementary Figure 46 Number of matched molecules for COMPACK clusters of 20 molecules for constrained optimisations using the Momany force field

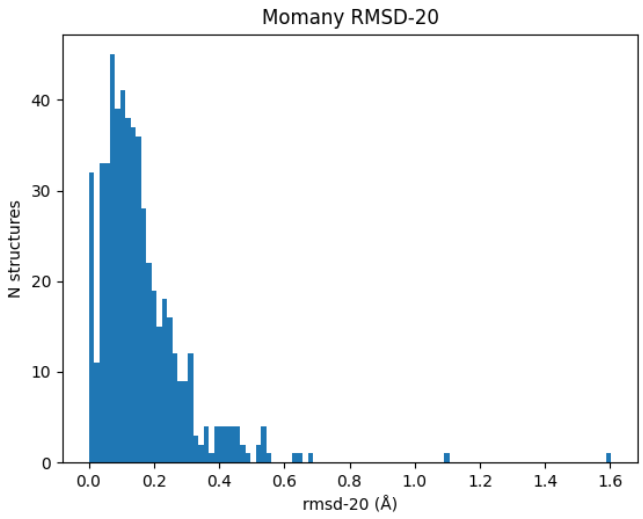

Supplementary Figure 47 RMSD-20 values (in Å) for COMPACK clusters of 20 molecules for constrained optimisations using the Momany force field

4.2.10 Crystal structures: Momany (full optimisation)

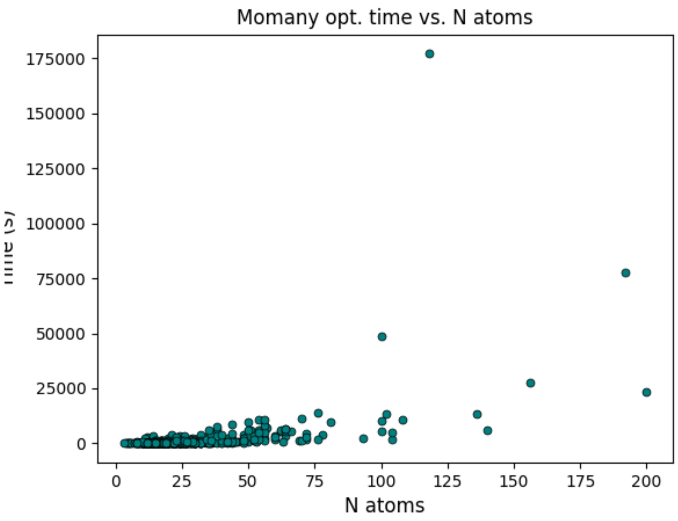

Supplementary Figure 48 Optimisation time vs. number of atoms for full optimisations using the Momany force field

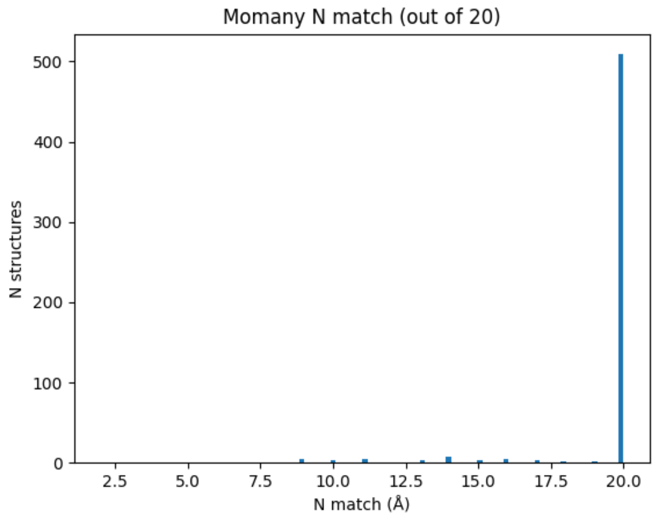

Supplementary Figure 49 Distribution of the number of matched molecules for COMPACK clusters of 20 molecules for full optimisations using the Momany force field

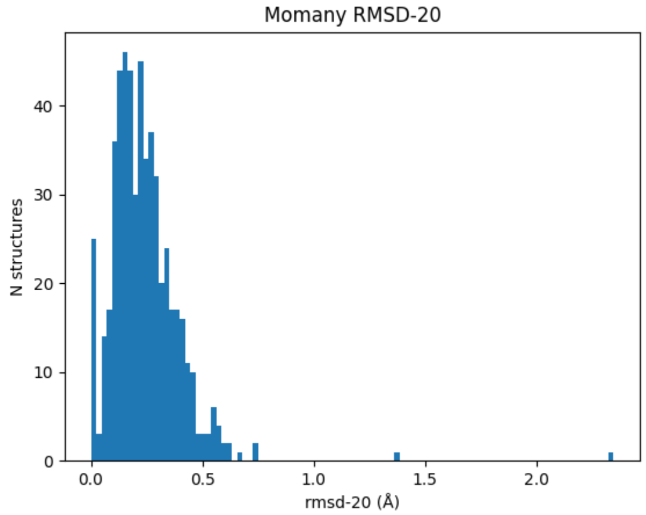

Supplementary Figure 50 Distribution of RMSD-20 values (in Å) for COMPACK clusters of 20 molecules for full optimisations using the Momany force field

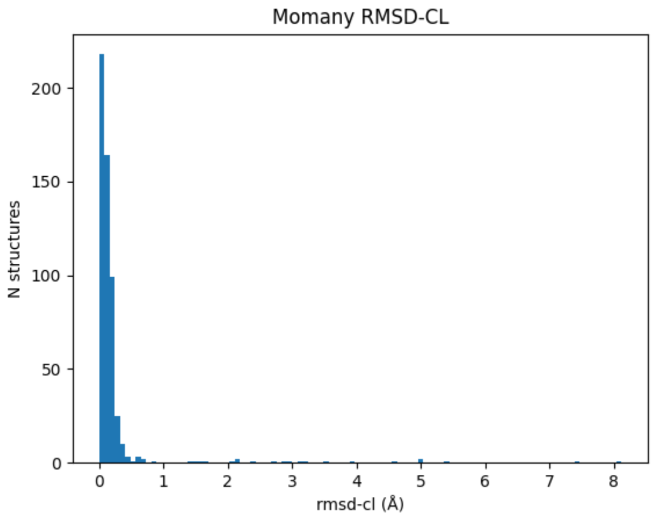

Supplementary Figure 51 Distribution of RMSD values for unit cell axes lengths (RMSD-CL, in Å) for full optimisations using the Momany force field

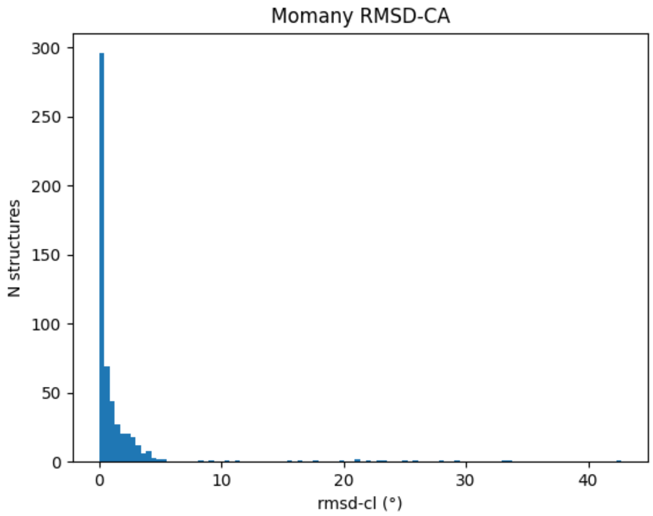

Supplementary Figure 52 Distribution of RMSD values for unit cell angles (RMSD-CA, in °) for full optimisations using the Momany force field

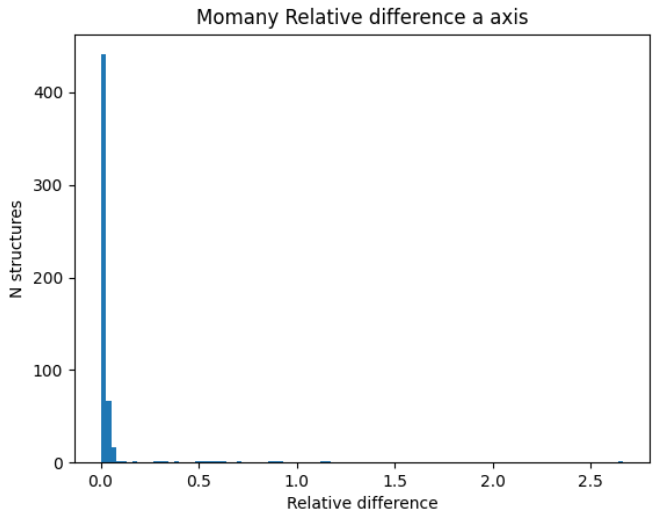

Supplementary Figure 53 Distribution of relative a axis values (with reference to corresponding CSD entry) for full optimisations using the Momany force field

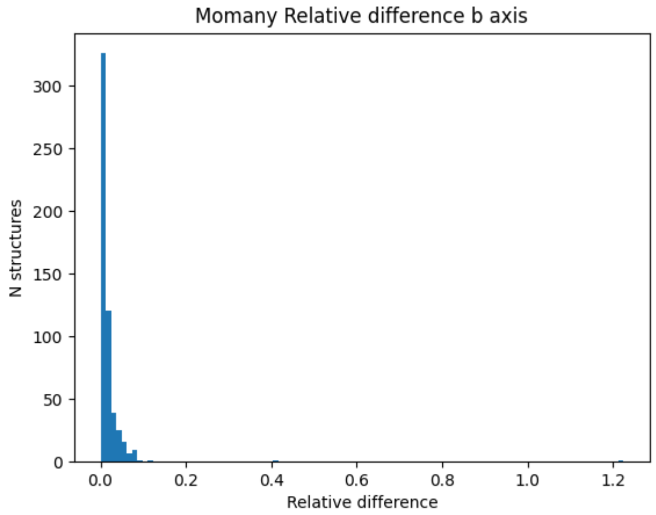

Supplementary Figure 54 Distribution of relative b axis values (with reference to corresponding CSD entry) for full optimisations using the Momany force field

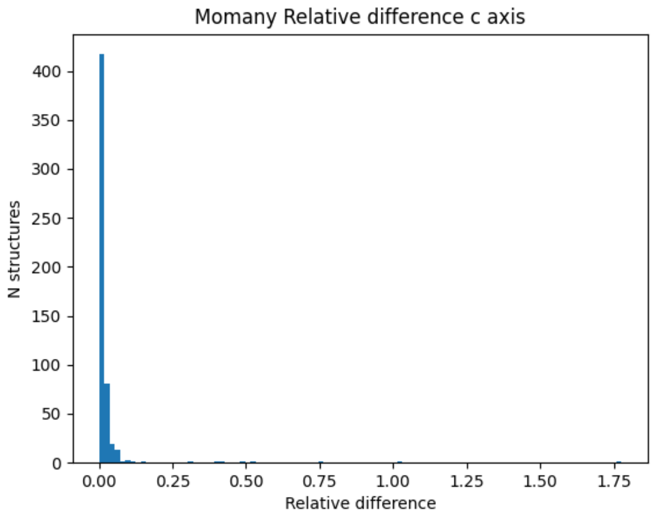

Supplementary Figure 55 Distribution of relative c axis values (with reference to corresponding CSD entry) for full optimisations using the Momany force field

4.2.11 Molecular geometries: CLP

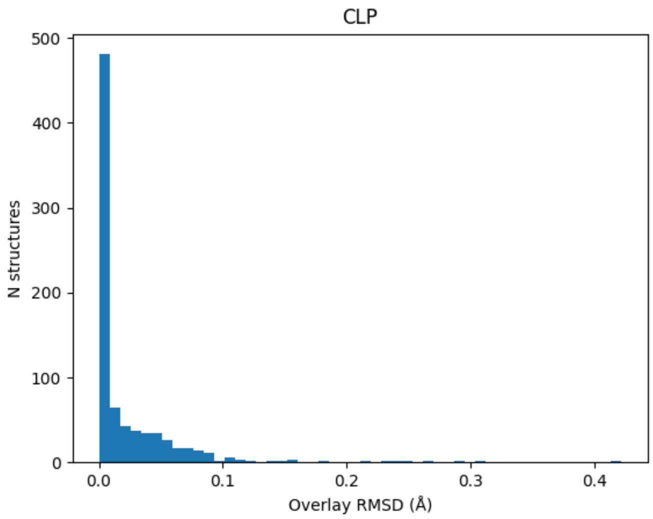

Supplementary Figure 56 Distribution of RMSD values for molecular overlays (RMSD-POS, in Å) in structures optimised with CLP compared to the corresponding CSD entry

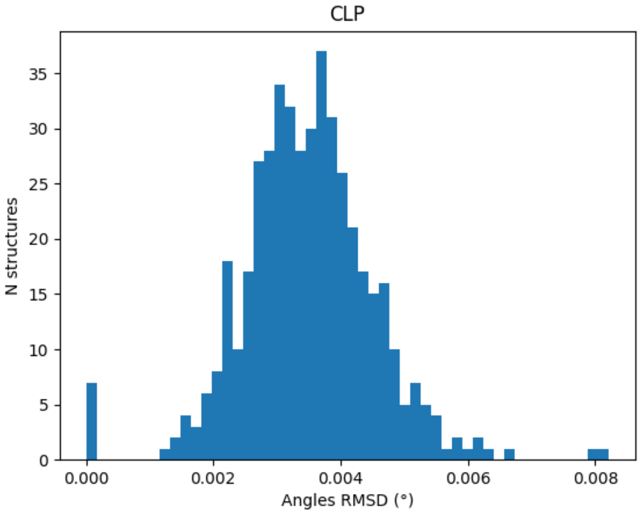

Supplementary Figure 57 Distribution of RMSD values for bond angles (RMSD-BA, in in °) in structures optimised with CLP compared to the corresponding CSD entry

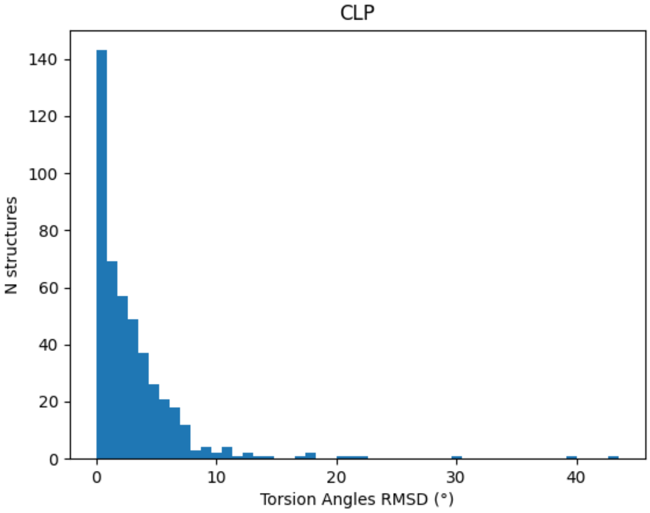

Supplementary Figure 58 Distribution of RMSD values for torsion angles (RMSD-TOR, in in °) in structures optimised with CLP compared to the corresponding CSD entry

4.2.12 Molecular geometries: UNI

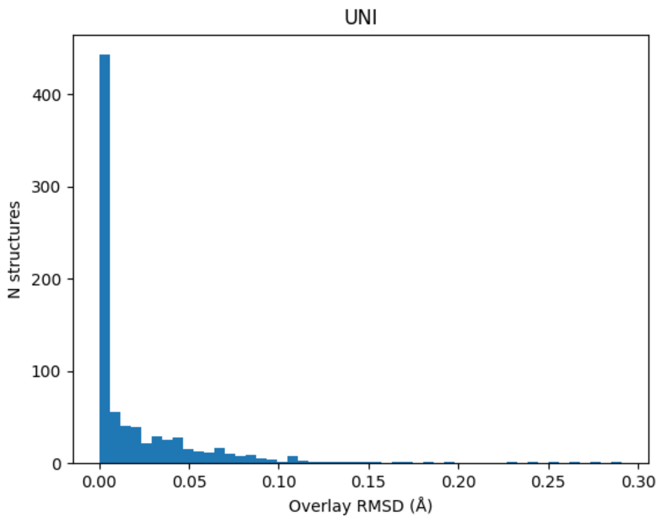

Supplementary Figure 59 Distribution of RMSD values for molecular overlays (RMSD-POS, in Å) in structures optimised with UNI compared to the corresponding CSD entry

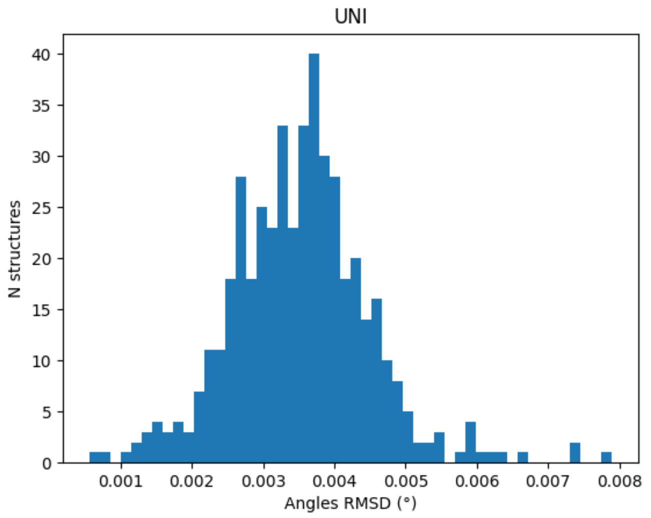

Supplementary Figure 60 Distribution of RMSD values for bond angles (RMSD-BA, in in °) in structures optimised with UNI compared to the corresponding CSD entry

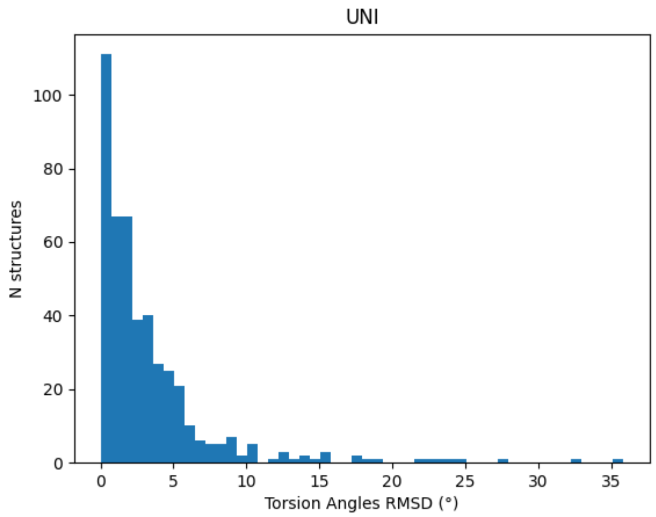

Supplementary Figure 61 Distribution of RMSD values for torsion angles (RMSD-TOR, in in °) in structures optimised with UNI compared to the corresponding CSD entry

4.2.13 Molecular geometries: CSD-OPCS16

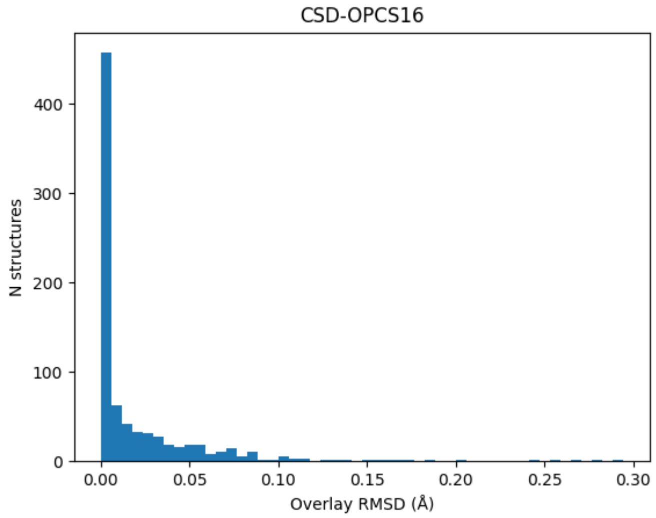

Supplementary Figure 62 Distribution of RMSD values for molecular overlays (RMSD-POS, in Å) in structures optimised with CSD-OPCS16 compared to the corresponding CSD entry

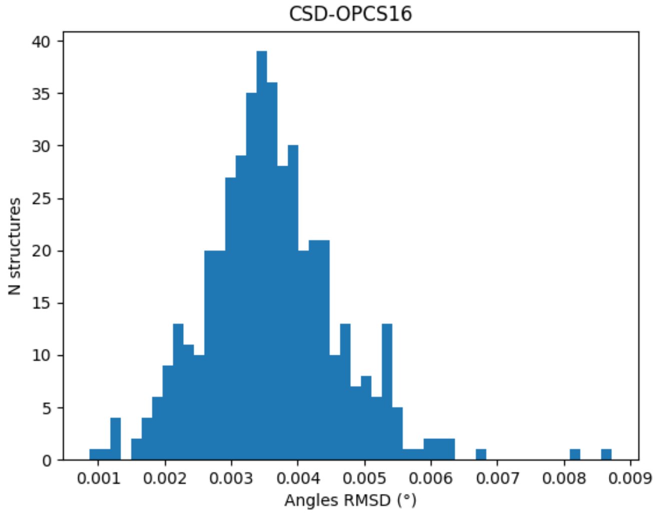

Supplementary Figure 63 Distribution of RMSD values for bond angles (RMSD-BA, in in °) in structures optimised with CSD-OPCS16 compared to the corresponding CSD entry

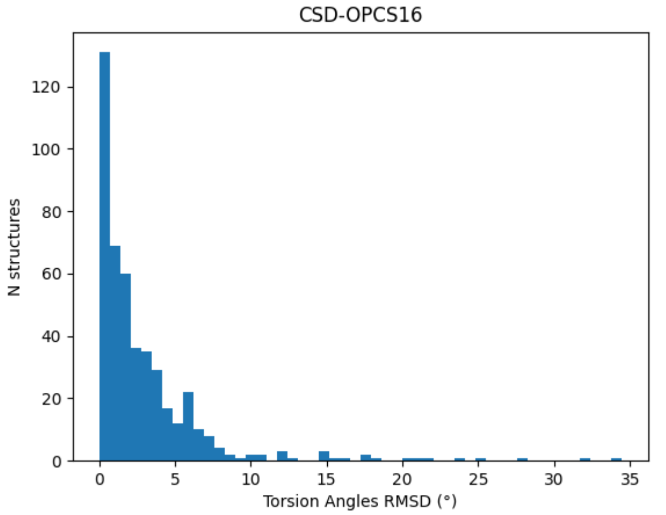

Supplementary Figure 64 Distribution of RMSD values for torsion angles (RMSD-TOR, in in °) in structures optimised with CSD-OPCS16 compared to the corresponding CSD entry

4.2.14 Molecular geometries: DreidingII

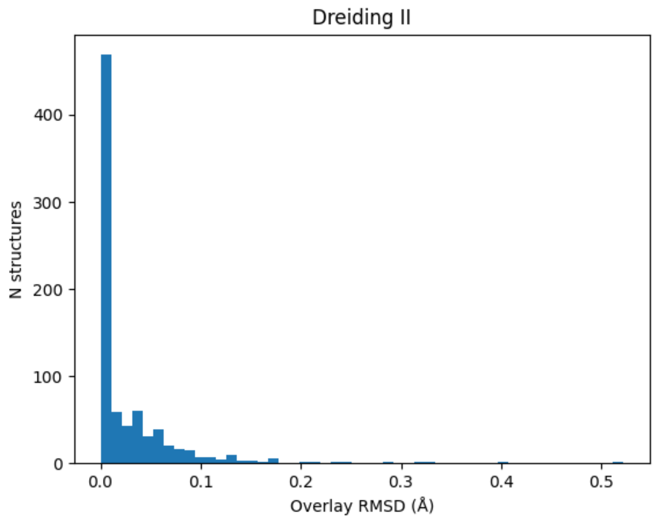

Supplementary Figure 65 Distribution of RMSD values for molecular overlays (RMSD-POS, in Å) in structures optimised with DreidingII compared to the corresponding CSD entry

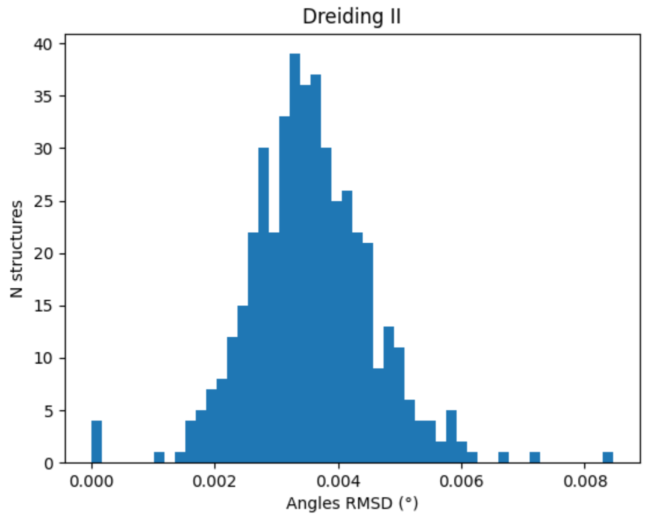

Supplementary Figure 66 Distribution of RMSD values for bond angles (RMSD-BA, in in °) in structures optimised with DreidingII compared to the corresponding CSD entry

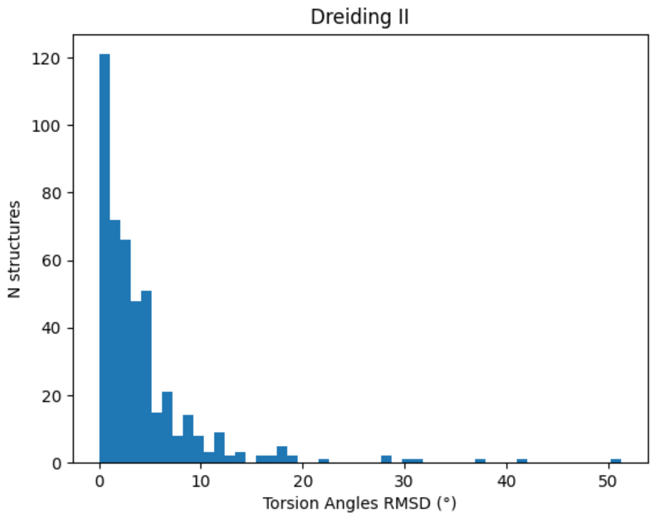

Supplementary Figure 67 Distribution of RMSD values for torsion angles (RMSD-TOR, in in °) in structures optimised with DreidingII compared to the corresponding CSD entry

4.2.15 Molecular geometries: Momany

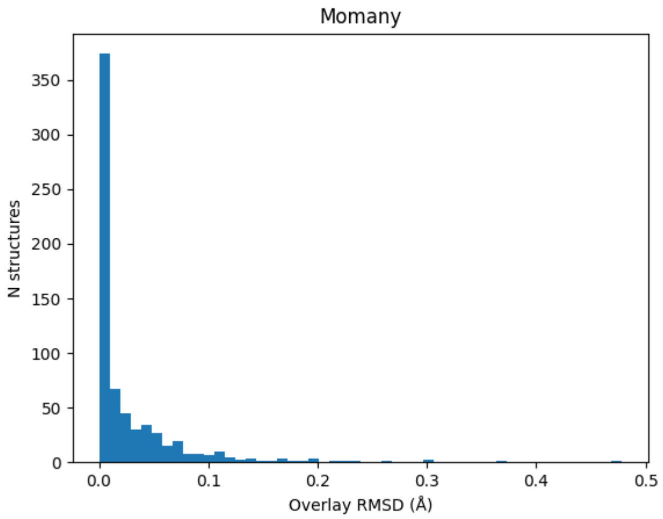

Supplementary Figure 68 Distribution of RMSD values for molecular overlays (RMSD-POS, in Å) in structures optimised with Momany compared to the corresponding CSD entry

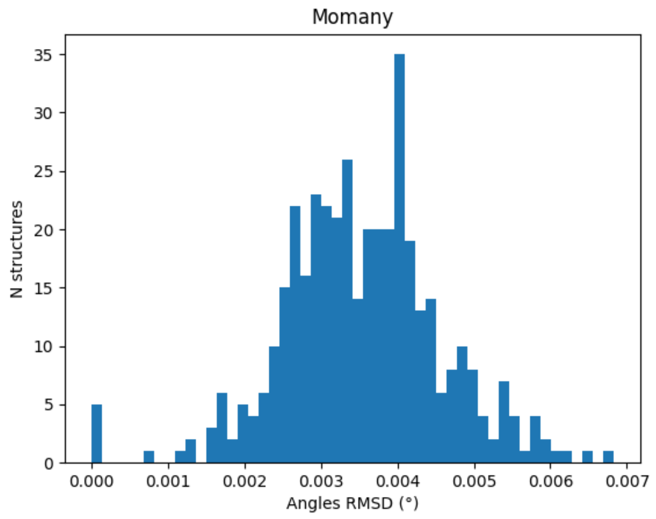

Supplementary Figure 69 Distribution of RMSD values for bond angles (RMSD-BA, in in °) in structures optimised with Momany compared to the corresponding CSD entry

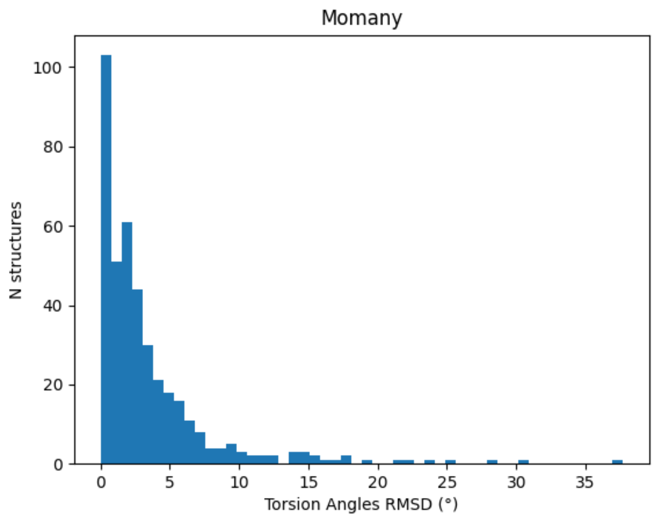

Supplementary Figure 70 Distribution of RMSD values for torsion angles (RMSD-TOR, in in °) in structures optimised with Momany compared to the corresponding CSD entry

5 Comparison of lattice energies with experimental sublimation enthalpies

5.1 Original CLP vs CSD-CLP

Lattice energies calculated with our implementation of the CLP force field (in *Visual-Habit* compared to results from from Chickos and Gavezzotti [1] for 663 entries show a mean absolute error of 1.8 kJ mol^{-1} and an RMSD of 3.2 kJ mol^{-1}, with avergare percentage error of 1.8%.

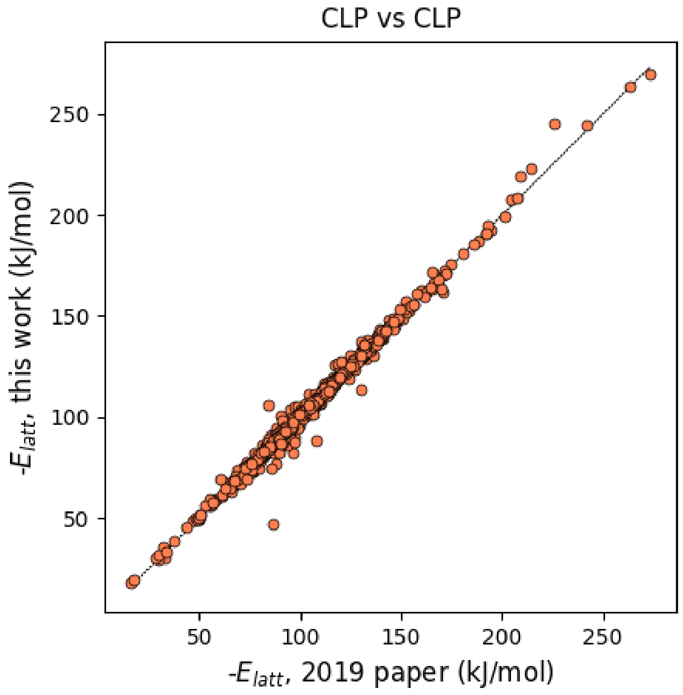

Supplementary Figure 71 Lattice energies for 663 compounds in the CG2019 dataset as calculated with our implementation of CLP vs. data from [1]

5.2 Lattice energy results by compound class

This section presents the results for the comparison of calculated lattice energies with experimental sublimation enthalpies divided by class of compound according to our classification (see main text for details).

Supplementary Table 7 Average error (%) for calculated lattice energies (CLP) in comparison to experimental sublimation enthalpies for different compound classes from the CG2019 dataset. Colours of cells are assigned based on values for each optimisation protocol. N is the number of structures in each category. Note that a single structure can be assigned to multiple categories.

CLP	N	No opt	X-H normalised	Constrained opt	Full opt
**ALL**	663	12.4	12.3	12.6	16.0
**Acid anhydride**	6	18.0	15.0	16.6	18.5
**Alcohol**	90	14.2	17.8	20.0	25.1
**Alkane**	210	13.4	13.2	14.1	16.8
**Alkene**	18	13.7	10.9	10.2	12.4
**Amide**	30	10.1	9.1	12.1	14.9
**Amine**	55	9.3	9.5	10.1	13.4
**Arene**	141	9.9	9.4	11.0	17.2
**Aromatic N**	74	10.8	10.9	10.3	12.8
**Carboxylic acid**	104	8.6	7.7	7.3	9.7
**Cyclic Ether**	13	8.7	7.2	7.8	10.7
**Ester**	15	14.9	10.6	12.8	15.6
**Ether**	22	8.5	6.9	6.2	8.1
**Haloalkane**	19	20.1	19.0	19.0	20.8
**Haloarene**	87	8.6	8.2	8.1	12.7
**Ketone**	45	16.0	15.1	17.5	21.0
**Nitrile**	16	12.0	11.1	12.1	17.7
**Nitro**	65	12.4	11.8	11.8	13.0
**Nucleic bases**	15	16.8	19.8	12.8	12.5
**Other**	17	12.5	13.2	14.0	17.3
**Other N**	10	19.2	21.3	17.0	21.6
**Other N O Cyclic**	24	13.4	14.5	11.5	13.8
**Other S**	22	18.6	18.8	18.4	21.2
**Sulfone**	6	9.8	7.3	7.6	10.5
**Urea Derivatives**	10	10.4	12.1	10.1	11.0

Supplementary Table 8 Average error (%) for calculated lattice energies (UNI) in comparison to experimental sublimation enthalpies for different compound classes from the CG2019 dataset. Colours of cells are assigned based on values for each optimisation protocol. N is the number of structures in each category. Note that a single structure can be assigned to multiple categories.

UNI	N	No opt	X-H normalised	Constrained opt	Full opt
**ALL**	664	11.7	10.4	10.4	11.7
**Acid anhydride**	6	21.0	20.6	17.4	17.1
**Alcohol**	90	11.1	10.7	10.9	13.9
**Alkane**	210	10.1	8.8	8.7	9.8
**Alkene**	18	12.2	7.9	6.9	6.6
**Amide**	30	10.4	8.7	8.8	9.1
**Amine**	55	10.9	11.1	10.9	12.7
**Arene**	141	11.0	9.0	9.6	11.1
**Aromatic N**	74	10.4	9.7	9.5	10.6
**Carboxylic acid**	104	9.7	6.3	6.4	8.0
**Cyclic Ether**	13	15.4	12.1	12.6	13.6
**Ester**	15	12.9	9.4	10.3	12.7
**Ether**	22	8.7	5.2	5.3	6.0
**Haloalkane**	19	21.4	20.9	20.4	20.9
**Haloarene**	87	8.8	7.9	8.3	10.5
**Ketone**	45	14.8	14.3	13.9	15.2
**Nitrile**	16	9.7	10.7	10.2	9.4
**Nitro**	65	12.4	11.7	12.8	16.1
**Nucleic bases**	15	14.1	13.5	12.1	11.2
**Other**	17	17.3	16.1	15.5	15.1
**Other N**	10	19.2	19.5	17.9	18.6
**Other N O Cyclic**	25	13.3	14.8	14.2	15.8
**Other S**	22	12.5	12.6	13.0	14.0
**Sulfone**	6	10.8	10.3	11.0	13.9
**Urea Derivatives**	10	9.1	6.9	6.7	6.9

Supplementary Table 9 Average error (%) for calculated lattice energies (CSD-OPCS16) in comparison to experimental sublimation enthalpies for different compound classes from the CG2019 dataset. Colours of cells are assigned based on values for each optimisation protocol. N is the number of structures in each category. Note that a single structure can be assigned to multiple categories.

CSD-OPCS16	N	No opt	X-H normalised	Constrained opt	Full opt
ALL	664	13.6	14.0	12.3	11.8
Acid anhydride	6	33.9	33.5	28.0	26.3
Alcohol	90	10.6	11.1	8.8	8.6
Alkane	210	10.8	12.3	10.9	10.2
Alkene	18	19.8	16.8	14.9	13.4
Amide	30	14.7	12.8	11.8	10.5
Amine	55	15.2	17.1	14.5	14.3
Arene	141	8.3	9.3	8.6	8.3
Aromatic N	74	12.7	12.3	10.5	10.2
Carboxylic acid	104	17.1	13.3	11.0	10.1
Cyclic Ether	13	18.6	16.0	14.6	13.8
Ester	15	7.6	7.4	6.5	6.4
Ether	22	16.1	13.2	11.3	10.2
Haloalkane	19	19.4	19.6	18.2	18.6
Haloarene	87	12.4	13.2	11.3	10.8
Ketone	45	16.9	17.3	15.1	14.3
Nitrile	16	15.6	17.3	15.9	15.0
Nitro	65	12.0	12.4	10.9	10.3
Nucleic bases	15	25.2	25.6	22.9	21.1
Other	17	17.1	16.4	15.5	13.7
Other N	10	21.9	22.3	20.2	19.8
Other N O Cyclic	25	20.4	22.3	19.8	20.5
Other S	22	14.7	15.4	14.6	14.5
Sulfone	6	7.3	8.5	8.1	8.0
Urea Derivatives	10	16.9	13.9	12.5	10.3

Supplementary Table 10 Average error (%) for calculated lattice energies (DreidingII) in comparison to experimental sublimation enthalpies for different compound classes from the CG2019 dataset. Colours of cells are assigned based on values for each optimisation protocol. N is the number of structures in each category. Note that a single structure can be assigned to multiple categories.

DreidingII	N	No opt	X-H normalised	Constrained opt	Full opt
ALL	664	25.8	27.1	17.7	15.8
Acid anhydride	6	21.5	22.9	19.9	19.8
Alcohol	90	22.3	21.1	11.5	12.5
Alkane	210	21.5	25.1	14.5	13.3
Alkene	18	44.8	39.1	20.8	14.3
Amide	30	15.3	23.2	18.9	14.8
Amine	55	19.0	25.6	21.0	20.5
Arene	141	18.1	13.9	10.0	10.1
Aromatic N	74	24.5	32.0	22.9	19.4
Carboxylic acid	104	68.4	59.5	18.8	12.5
Cyclic Ether	13	62.4	33.9	21.6	19.5
Ester	15	10.1	18.6	15.4	9.3
Ether	22	56.4	42.6	16.2	9.9
Haloalkane	19	31.0	32.2	26.7	26.8
Haloarene	87	22.4	22.1	15.5	15.2
Ketone	45	16.5	21.4	16.5	14.4
Nitrile	16	21.5	28.3	24.9	20.5
Nitro	65	21.2	28.0	22.1	19.7
Nucleic bases	15	36.6	48.5	39.1	31.5
Other	17	15.6	20.1	14.2	11.9
Other N	10	30.4	40.8	33.3	29.6
Other N O Cyclic	25	19.9	29.6	22.3	19.7
Other S	22	23.0	26.7	22.3	19.5
Sulfone	6	11.5	15.1	13.3	12.3
Urea Derivatives	10	21.9	32.1	29.2	27.1

Supplementary Table 11 Average error (%) for calculated lattice energies (Momany) in comparison to experimental sublimation enthalpies for different compound classes from the CG2019 dataset. Colours of cells are assigned based on values for each optimisation protocol.

Momany	N	No opt	X-H normalised	Constrained opt	Full opt
**ALL**	550	28.4	28.5	23.6	21.7
**Acid anhydride**	6	17.8	18.6	16.7	16.4
**Alcohol**	80	25.3	20.0	12.7	11.3
**Alkane**	206	19.0	21.1	17.7	16.3
**Alkene**	18	40.3	32.9	27.9	23.5
**Amide**	29	32.5	33.2	29.7	27.4
**Amine**	40	37.1	42.0	34.1	32.0
**Arene**	134	17.5	18.1	15.8	14.2
**Aromatic N**	63	52.8	57.1	42.2	39.2
**Carboxylic acid**	90	33.4	18.5	11.2	8.9
**Cyclic Ether**	12	29.6	16.5	13.2	10.7
**Ester**	15	9.7	13.7	11.4	8.4
**Ether**	21	33.0	16.7	12.7	9.9
**Haloalkane**	-	-	-	-	-
**Haloarene**	-	-	-	-	-
**Ketone**	40	19.8	22.1	19.1	18.7
**Nitrile**	16	50.3	57.7	52.7	46.1
**Nitro**	48	21.5	23.8	19.3	18.1
**Nucleic bases**	11	53.0	56.6	50.6	48.5
**Other**	17	26.4	30.3	25.0	23.2
**Other N**	10	63.6	69.5	60.9	57.0
**Other N O Cyclic**	25	32.2	39.1	31.5	29.9
**Other S**	22	59.2	61.6	57.9	56.5
**Sulfone**	6	30.6	35.3	33.0	32.1
**Urea Derivatives**	10	38.7	39.4	37.1	36.2

6 Polymorphic dataset

6.1 Ritonavir

Supplementary Table 12 Results (number of molecules matched and RMSD from structure overlay) from COMPACK comparisons (distance and angle tolerances of 35% and 35° for a cluster of 30 molecules) of the experimental versus geometry-optimised crystal structures of Ritonavir polymorphs for each force field.

**Polymorph**	**CSD REFCODE**	CLP		DreidingII		UNI		Momany	
		Mol.	RMSD	Mol.	RMSD	Mol.	RMSD	Mol.	RMSD
I	YIGPIO02	30	0.526	30	0.406	30	0.297	30	0.381
II	YIGPIO03	30	0.000	30	0.390	30	0.202	30	0.280

Supplementary Table 13 Ritonavir: Absolute energies of each polymorph, after full geometry optimisation, calculated at PBE-GD2 and PBE-TS levels of theory (literature values), and using the CLP, DreidingII, UNI, and Momany force fields.

Polymorph	CSD refcode	Absolute energy (kJ/mol)					
		PBE-GD2	PBE-TS	CLP	Dreiding	UNI	Momany
I	YIGPIO02	-383.9	-456.8	-371.9	-307.6	-369.4	-221.4
II	YIGPIO03	-423.0	-491.2	-393.4	-331.3	-390.1	-258.0

Supplementary Table 14 Ritonavir: Relative energies of each polymorph, after full geometry optimisation, calculated at PBE-GD2 and PBE-TS levels of theory (literature values), and using the CLP, DreidingII, UNI, and Momany force fields.

Polymorph	CSD refcode	Relative energy (kJ/mol)					
		PBE-GD2	PBE-TS	CLP	Dreiding	UNI	Momany
I	YIGPIO02	39.10	34.40	21.5	23.7	20.7	36.6
II	YIGPIO03	0.00	0.00	0.0	0.0	0.0	0.0

6.2 ROY

Supplementary Table 15 Results (number of molecules matched and RMSD from structure overlay) from COMPACK comparisons (distance and angle tolerances of 35% and 35° for a cluster of 30 molecules) of the experimental versus geometry-optimised crystal structures of ROY polymorphs for each force field.

**Polymorph**	**CSD REFCODE**	CLP		DreidingII		UNI		Momany	
		Mol.	RMSD	Mol.	RMSD	Mol.	RMSD	Mol.	RMSD
Y	QAXMEH22	30	0.492	30	0.221	30	0.321	30	0.128
YT	QAXMEH12	30	0.368	30	0.297	30	0.306	30	0.234
R	QAXMEH02	30	0.254	30	0.401	30	0.265	30	0.299
OP	QAXMEH03	30	0.347	30	0.065	30	0.321	30	0.254
YN	QAXMEH04	30	0.452	30	0.641	30	0.295	30	0.313
Y0	QAXMEH53	30	0.326	30	0.386	30	0.340	26	0.637
R0	QAXMEH31	30	0.439	30	0.358	30	0.333	30	0.387
PO	QAXMEH52	30	0.485	30	0.187	30	0.300	30	0.265
ON	QAXMEH	30	0.719	30	0.381	30	0.395	30	0.463
OR	QAXMEH05	30	0.450	30	0.156	30	0.395	30	0.281
R1	QAXMEH57	30	0.668	30	0.652	30	0.284	30	0.905
Y1	QAXMEH60	30	0.558	30	0.383	30	0.296	30	0.389

Supplementary Table 16 ROY: Absolute energies of each polymorph, after full geometry optimisation, calculated at B86bPBE-XDM and SCS-MP2D levels of theory (literature values), and using the CLP, DreidingII, UNI, and Momany force fields.

Polymorph	CSD refcode	Absolute energy (kJ/mol)				
		SCS-MP2D	CLP	DreidingII	UNI	Momany
Y	QAXMEH22	-128.25	-165.1	-122.068	-176.3	-84.5
YT04	QAXMEH12	-127.61	-169.8	-122.934	-168.8	-84.9
R	QAXMEH02	-127.42	-163.2	-125.041	-156.4	-85.3
OP	QAXMEH03	-126.64	-169.1	-114.704	-169.4	-82.5
YN	QAXMEH04	-125.94	-169.9	-123.145	-153.2	-85.5
Y04	QAXMEH53	-125.83	-174.7	-114.977	-163.3	-81.1
R05	QAXMEH31	-125.64	-179.8	-113.465	-155.5	-76.1
PO13	QAXMEH52	-125.45	-158.7	-122.064	-163.5	-84.8
ON	QAXMEH	-125.23	-142.7	-117.959	-160.0	-83.9
ORP	QAXMEH05	-123.96	-156.4	-115.562	-155.8	-81.6
R18	QAXMEH57	-123.39	-164.2	-114.295	-157.6	-77.4
Y19	QAXMEH60	-121.64	-139.3	-122.057	-162.4	-87.6

Supplementary Table 17 ROY: Relative energies of each polymorph, after full geometry optimisation, calculated at B86bPBE-XDM and SCS-MP2D levels of theory (literature values), and using the CLP, DreidingII, UNI, and Momany force fields.

Polymorph	CSD refcode	Relative energy (kJ/mol)				
		SCS-MP2D	CLP	DreidingII	UNI	Momany
Y	QAXMEH22	0	14.7	3.0	0.0	3.1
YT04	QAXMEH12	0.64	10.0	2.1	7.5	2.7
R	QAXMEH02	0.83	16.6	0.0	19.9	2.3
OP	QAXMEH03	1.61	10.7	10.3	6.9	5.1
YN	QAXMEH04	2.31	9.9	1.9	23.1	2.0
Y04	QAXMEH53	2.42	5.0	10.1	13.0	6.5
R05	QAXMEH31	2.61	0.0	11.6	20.8	11.5
PO13	QAXMEH52	2.8	21.1	3.0	12.8	2.8
ON	QAXMEH	3.02	37.1	7.1	16.3	3.7
ORP	QAXMEH05	4.29	23.4	9.5	20.5	6.0
R18	QAXMEH57	4.86	15.6	10.7	18.7	10.2
Y19	QAXMEH60	6.61	40.5	3.0	13.9	0.0

Supplementary Table 18 ROY: Rank order of each polymorph, after full geometry optimisation, calculated at B86bPBE-XDM and SCS-MP2D levels of theory (literature values), and using the CLP, DreidingII, UNI, and Momany force fields.

Polymorph	CSD refcode	Rank order				
		SCS-MP2D	CLP	DreidingII	UNI	Momany
Y	QAXMEH22	1	6	4	1	6
YT04	QAXMEH12	2	4	3	3	4
R	QAXMEH02	3	8	1	9	3
OP	QAXMEH03	4	5	10	2	8
YN	QAXMEH04	5	3	2	12	2
Y04	QAXMEH53	6	2	9	5	10
R05	QAXMEH31	7	1	12	11	12
PO13	QAXMEH52	8	9	5	4	5
ON	QAXMEH	9	11	7	7	7
ORP	QAXMEH05	10	10	8	10	9
R18	QAXMEH57	11	7	11	8	11
Y19	QAXMEH60	12	12	6	6	1

6.3 Tolfenamic acid (TFA)

Supplementary Table 19 Results (number of molecules matched and RMSD from structure overlay) from COMPACK comparisons (distance and angle tolerances of 35% and 35° for a cluster of 30 molecules) of the experimental versus geometry-optimised crystal structures of TFA polymorphs for each force field.

**Polymorph**	**CSD REFCODE**	CLP		DreidingII		UNI	
		Mol.	RMSD	Mol.	RMSD	Mol.	RMSD
I	KAXXAI01	30	0.287	30	0.393	30	0.150
II	KAXXAI	30	0.223	30	0.300	30	0.119
III	KAXXAI02	30	0.272	30	0.220	30	0.132
IV	KAXXAI03	30	0.000	30	0.000	30	0.239
V	KAXXAI04	30	0.079	30	0.271	30	0.270
VI	KAXXAI08	30	0.318	30	0.264	30	0.139
VII	KAXXAI05	30	0.334	30	0.154	30	0.204
VIII	KAXXAI06	30	0.254	30	0.958	30	0.967
IX	KAXXAI11	30	0.254	30	0.231	30	0.204

Supplementary Table 20 Additional COMPACK comparison (distance and angle tolerances of 35% and 35° for a cluster of 30 molecules) results (number of molecules matched and RMSD from structure overlay) of the experimental and geometry-optimised crystal structures of polymorphs IV (KAXXAI03) and VI (KAXXAI08) of TFA for each force field.

**CSD REFCODE**	**Optimised structure**	CLP		DreidingII		UNI	
		Mol.	RMSD	Mol.	RMSD	Mol.	RMSD
KAXXAI03	Optimised_KAXXAI08	30	0.493	30	0.479	30	0.391
KAXXAI08	Optimised_KAXXAI03	30	0.379	30	0.379	30	0.354

Supplementary Table 21 TFA: Absolute energies of each polymorph, after full geometry optimisation, calculated at the PBE-MBD level of theory (literature values), and using the CLP, DreidingII, and UNI force fields.

Polymorph	CSD refcode	Absolute energy (kJ/mol)			
		PBE-MBD	CLP	Dreiding	UNI
II	KAXXAI	-158.5	-153.8	-139.6	-160.1
IX	KAXXAI11	-157.2	-151.7	-136.5	-159.6
I	KAXXAI01	-154.3	-148.5	-130.7	-157.0
IV	KAXXAI03	-153.5	-143.1	-77.5	-159.4
VI	KAXXAI08	-153.4	-151.5	-107.7	-159.6
III	KAXXAI02	-153.3	-155.1	-136.5	-160.0
VIII	KAXXAI06	-151.8	-138.4	-129.6	-154.7
VII	KAXXAI05	-151.7	-144.9	-127.2	-151.5
V	KAXXAI04	-147.6	-139.1	-134.9	-160.8

Supplementary Table 22 TFA: Relative energies of each polymorph, after full geometry optimisation, calculated at the PBE-MBD level of theory (literature values), and using the CLP, DreidingII, and UNI force fields.

Polymorph	CSD refcode	Relative energy (kJ/mol)			
		PBE-MBD	CLP	Dreiding	UNI
II	KAXXAI	0.00	1.3	0.0	0.7
IX	KAXXAI11	1.32	3.4	3.1	1.2
I	KAXXAI01	4.21	6.6	8.9	3.8
IV	KAXXAI03	5.07	11.9	62.1	1.5
VI	KAXXAI08	5.11	3.6	31.9	1.3
III	KAXXAI02	5.26	0.0	3.1	0.9
VIII	KAXXAI06	6.67	16.7	10.0	6.2
VII	KAXXAI05	6.86	10.2	12.4	9.3
V	KAXXAI04	10.95	16.0	4.7	0.0

Supplementary Table 23 TFA: Rank order of each polymorph, after full geometry optimisation, calculated at the PBE-MBD level of theory (literature values), and using the CLP, DreidingII, and UNI force fields.

Polymorph	CSD refcode	Rank			
		PBE-MBD	CLP	Dreiding	UNI
II	KAXXAI	1	2	1	2
IX	KAXXAI11	2	3	3	4
I	KAXXAI01	3	5	5	7
IV	KAXXAI03	4	7	9	6
VI	KAXXAI08	5	4	8	5
III	KAXXAI02	6	1	2	3
VIII	KAXXAI06	7	9	6	8
VII	KAXXAI05	8	6	7	9
V	KAXXAI04	9	8	4	1

6.4 PF-06282999

Supplementary Table 24 Results (number of molecules matched and RMSD from structure overlay) from COMPACK comparisons (distance and angle tolerances of 35% and 35° for a cluster of 30 molecules) of the experimental versus geometry-optimised crystal structures of PF-06282999 polymorphs for each force field.

**Polymorph**	**CSD refcode**	CLP		DreidingII		UNI	
		Mol.	RMSD	Mol.	RMSD	Mol.	RMSD
1	XULBAL01	30	0.440	30	0.181	30	0.433
2	XULBAL03	30	0.286	30	0.321	30	0.144
3	XULBAL02	30	0.478	30	0.281	30	0.312
4	XULBAL	30	0.636	30	0.161	30	0.320

Supplementary Table 25 Absolute energies of each polymorph, after full geometry optimisation, calculated using the CLP, DreidingII, and UNI force fields for PF-06282999 polymorphs

Polymorph	CSD refcode	Absolute energy (kJ/mol)		
		CLP	DreidingII	UNI
3	XULBAL02	-198.4	-171.1	-201.5
1	XULBAL01	-204.1	-173.1	-222.2
2	XULBAL03	-194.7	-145.7	-197.1
4	XULBAL	-187.6	-164.0	-198.6

Supplementary Table 26 MPO: Relative energies of each polymorph, after full geometry optimisation, calculated at the PBE-TS and optPBE-vdw levels of theory (literature values), and using the CLP, DreidingII, and UNI force fields.

Polymorph	CSD refcode	Relative energy (kJ/mol)				
		PBE-TS	optPBE-vdW	CLP	DreidingII	UNI
3	XULBAL02	0	0	5.7	2.0	20.6
1	XULBAL01	0.4	1.2	0.0	0.0	0.0
2	XULBAL03	2.5	0.8	9.3	27.4	25.1
4	XULBAL	10.4	12.4	16.5	9.1	23.6

Supplementary Table 27 Rank order of each polymorph, after full geometry optimisation, calculated at the PBE-TS and optPBE-vdw levels of theory (literature values), and using the CLP, DreidingII, and UNI force fields for PF-06282999 polymorphs.

Polymorph	CSD refcode	Rank				
		PBE-TS	optPBE-vdW	CLP	DreidingII	UNI
3	XULBAL02	1	1	2	2	2
1	XULBAL01	2	3	1	1	1
2	XULBAL03	3	2	3	4	4
4	XULBAL	4	4	4	3	3

References

[1] Chickos, J.S., Gavezzotti, A.: Sublimation enthalpies of organic compounds: a very large database with a match to crystal structure determinations and a comparison with lattice energies. Crystal Growth & Design **19**(11), 6566–6576 (2019)

[2] Gavezzotti, A.: Efficient computer modeling of organic materials. the atom–atom, coulomb–london–pauli (aa-clp) model for intermolecular electrostatic-polarization, dispersion and repulsion energies. New Journal of Chemistry **35**(7), 1360–1368 (2011) https://doi.org/10.1039/C0NJ00982B

[3] Hoffmann, R.: An extended hückel theory. i. hydrocarbons. The Journal of Chemical Physics **39**(6), 1397–1412 (1963) https://doi.org/10.1063/1.1734456 https://pubs.aip.org/aip/jcp/article-pdf/39/6/1397/18830291/1397$_1$$_o$*nline.pdf*

[4] Gavezzotti, A., Presti, L.L., Rizzato, S.: Molecular dynamics simulation of organic materials: structure, potentials and the micmos computer platform. CrystEngComm **24**, 922–930 (2022) https://doi.org/10.1039/D1CE01360B

[5] Filippini, G., Gavezzotti, A.: Empirical intermolecular potentials for organic crystals: the '6-exp' approximation revisited. Acta Crystallographica Section B **49**(5), 868–880 (1993) https://doi.org/10.1107/S0108768193002150

[6] Gavezzotti, A.: Are crystal structures predictable? Accounts of chemical research **27**(10), 309–314 (1994) https://doi.org/10.1021/ar00046a004

[7] Gavezzotti, A., Filippini, G.: Geometry of the intermolecular xh. cntdot.. cntdot.. cntdot. y (x, y= n, o) hydrogen bond and the calibration of empirical hydrogen-bond potentials. The Journal of Physical Chemistry **98**(18), 4831–4837 (1994) https://doi.org/10.1021/j100069a010

[8] Cole, J.C., Groom, C.R., Read, M.G., Giangreco, I., McCabe, P., Reilly, A.M., Shields, G.P.: Generation of crystal structures using known crystal structures as analogues. Acta Crystallographica Section B **72**(4), 530–541 (2016) https://doi.org/10.1107/S2052520616006533

[9] Mayo, S.L., Olafson, B.D., Goddard, W.A.: Dreiding: a generic force field for molecular simulations. Journal of Physical chemistry **94**(26), 8897–8909 (1990) https://doi.org/10.1021/j100389a010

[10] Momany, F., Carruthers, L., McGuire, R.t., Scheraga, H.: Intermolecular potentials from crystal data. iii. determination of empirical potentials and application to the packing configurations and lattice energies in crystals of hydrocarbons, carboxylic acids, amines, and amides. The Journal of Physical Chemistry **78**(16), 1595–1620 (1974) https://doi.org/10.1021/j100609a005

[11] Gasteiger, J., Marsili, M.: A new model for calculating atomic charges in molecules. Tetrahedron Letters **19**(34), 3181–3184 (1978) https://doi.org/10.1016/S0040-4039(01)94977-9

[12] Chisholm, J.A., Motherwell, S.: *COMPACK*: a program for identifying crystal structure similarity using distances. Journal of Applied Crystallography **38**(1), 228–231 (2005) https://doi.org/10.1107/S0021889804027074

[13] Sacchi, P., Lusi, M., Cruz-Cabeza, A.J., Nauha, E., Bernstein, J.: Same or different – that is the question: identification of crystal forms from crystal structure data. CrystEngComm **22**, 7170–7185 (2020) https://doi.org/10.1039/D0CE00724B
